# Research on Memristors in Hardware Security

**DOI:** 10.3390/mi17091040

**Published:** 2026-08-31

**Authors:** Zihan Zhong, Chenqi Dai, Guangfu Luo, Yaoyao Jin, Xuenan Peng, Tao Wang, Shuai Zhang, Cong Ye

**Affiliations:** 1 International School of Integrated Circuit, Dongguan University of Technology, Dongguan 523000, China; m19330234709@163.com (Z.Z.); daichenqi1217@outlook.com (C.D.); romate0306@gmail.com (G.L.); xtunpxn@163.com (X.P.); 2025084@dgut.edu.cn (T.W.); 2 Key Laboratory of Intelligent Sensing System and Security of the Ministry of Education, School of Microelectronics, Hubei University, Wuhan 430062, China; jyy1298758616@163.com

**Keywords:** memristors, true random number generators, physical unclonable functions, hardware security

## Abstract

As the deep integration of the Internet of Things (IoT) and artificial intelligence (AI) technologies has rendered traditional encryption techniques increasingly inadequate in terms of security strength, the implementation of hardware-level security solutions has been identified as a critical issue in the design of information systems. Memristors, as passive circuit components with their intrinsic stochastic switching behavior, multistate storage capability, and low power consumption, have been exploited to provide a viable approach for the construction of hardware security primitives, including true random number generators (TRNGs) and physically unclonable functions (PUFs). In this review, the latest research advances in memristor-based hardware security technologies were systematically surveyed from three perspectives, namely TRNGs, PUFs and other security schemes. With regard to TRNGs and PUFs, respectively, we have systematically summarized their characteristics, including throughput, power consumption and randomness quality, as well as uniqueness, reliability and methods, with particular attention paid to the types of entropy sources and bit-generation strategies. In addition, the potential application of other memristor-based hardware security solutions in information hiding, encrypted transmission and authentication was also analyzed. Subsequently, the principal challenges impeding the transition from laboratory research to mass production were summarized, encompassing synergistic integration with advanced CMOS processes, the balance between reliability and stochasticity, and the absence of design methodologies that integrate hardware and software. Future development trajectories were further delineated, including cross-layer optimization across devices, circuits, and systems, the design of high-security chips resistant to machine learning attacks, CMOS-compatible stable implementation schemes, and the on-chip integration of TRNGs, PUFs, and encryption engines. The present review is intended to serve as a systematic reference for the design of novel hardware security systems, thereby paving the way for the practical deployment of memristor-based security technologies.

## 1. Introduction

In contemporary society, characterized by the deep integration of digitization, intelligent technologies and networking, the rapid development of technologies such as the IoT, edge computing and AI is reshaping human life and industrial structures in unprecedented ways [1,2,3]. The massive deployment of smart devices and sensors has enabled seamless interconnection and real-time interaction between the physical and digital worlds, fueling a surge in data generation that is projected to reach 175 ZB globally by 2025, with over 30% of this volume demanding real-time or near-real-time processing [4,5,6]. Consequently, conventional von Neumann computing, characterized by the separation of the central processing unit and memory, faces severe challenges, namely the well-recognized ‘memory wall’ and ‘power wall’ bottlenecks [7,8,9,10,11]. Compounding these challenges, the escalating value of data has elevated information security and privacy protection to core bottlenecks that increasingly constrain the development of the digital economy [12]. Traditional software encryption algorithms, such as the Advanced Encryption Standard (AES) and Rivest–Shamir–Adleman (RSA), offer high theoretical security. In practice, their key generation often relies on cryptographically secure pseudorandom number generators (CSPRNGs). Although CSPRNGs are highly robust when using seeds with sufficient entropy, the mathematical foundations of these algorithms are increasingly under threat from attackers with powerful computing capabilities, following the emergence of quantum computing and machine learning-based cryptanalytic techniques. System security is further compromised by software vulnerabilities, backdoors, and side-channel attacks [13,14,15].

Against this background, hardware security primitives have emerged as the cornerstone for building next-generation trusted computing systems. Diverging fundamentally from software solutions, these primitives derive their security services from the inherent randomness, unpredictability, and unclonability of microscopic physical processes, thereby hardening the system against attacks at its very foundation [16,17]. Among them, true random number generators serve as the core components for generating cryptographic keys and random seeds, with the quality of their randomness directly dictating the security strength of the entire cryptographic infrastructure [18,19,20,21]. Meanwhile, the physical unclonable function utilizes microscopic physical variations introduced during the manufacturing process that are impossible to replicate, providing devices with a unique ‘digital fingerprint’; such functionality is widely applied in fields such as chip authentication, anti-counterfeiting, and traceability [22,23,24,25].

**Figure 1:**
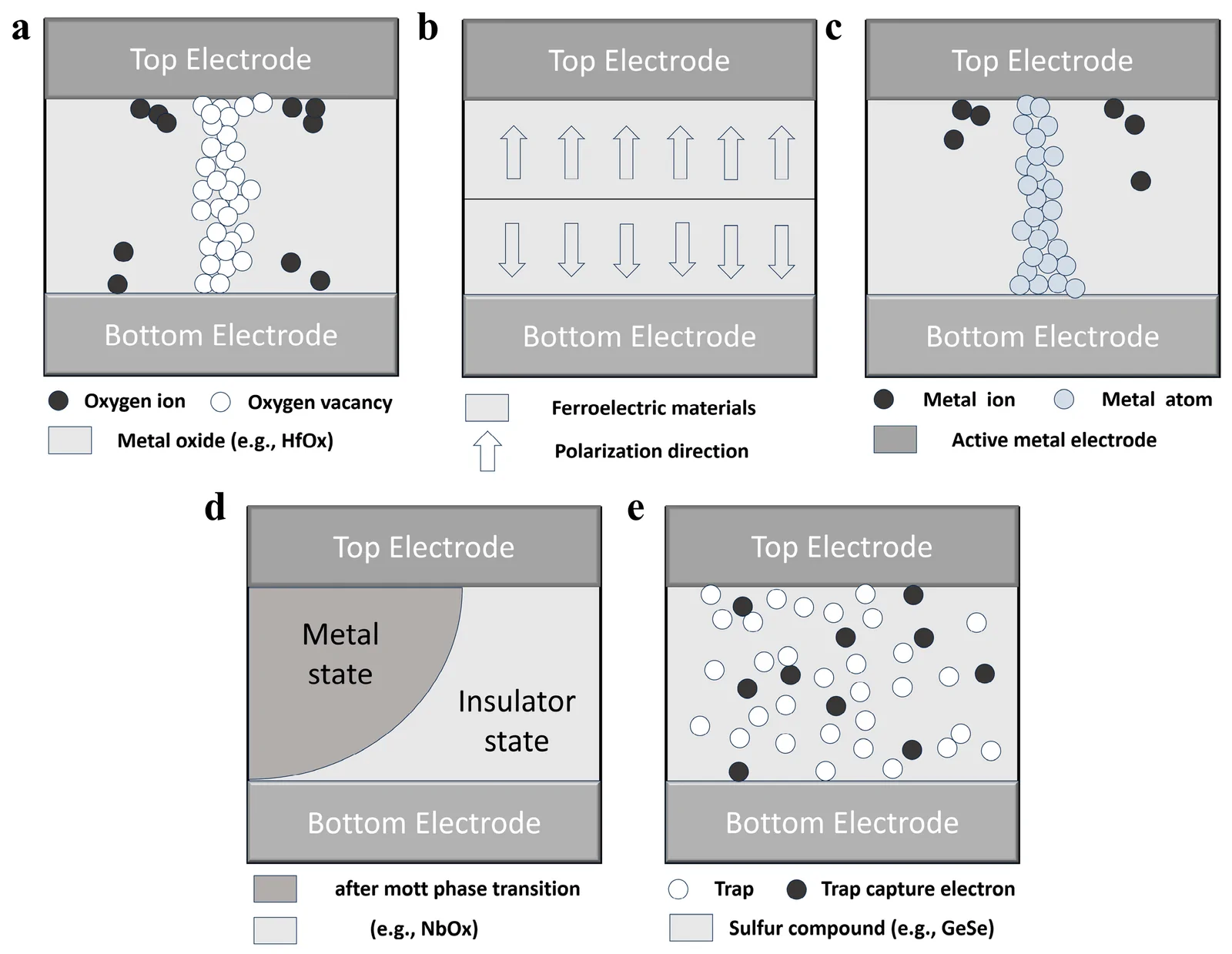
Schematic diagram of different device switching mechanisms: (**a**) resistive random-access memory, (**b**) ferroelectric memristors, (**c**) diffusive memristor, (**d**) Mott memristor, and (**e**) OTS devices.

In 1971, Chua first proposed the concept of the ideal memristor, defining it as the fourth fundamental two-terminal circuit element alongside the resistor, capacitor, and inductor. Its constitutive relationship is determined by the charge and magnetic flux φ, with the memristor value depending solely on the total charge that has flowed through the device [26]. However, the ideal memristor is a theoretical construct subject to stringent physical constraints, and no practically realizable device perfectly satisfies this definition. To accommodate physical devices, Chua and Kang generalized the concept to memristive systems in 1976 [27]. A memristive system is a broader class of dynamical systems that exhibit pinched hysteresis loops in their current–voltage characteristics under periodic excitation, but whose state variables may depend on more than just charge or flux. Consequently, most practical resistive-switching devices are more accurately classified as memristive systems rather than ideal memristors. As shown in Figure 1, depending on whether the resistance state is retained after the removal of the applied bias, memristive systems can be divided into two main categories: non-volatile and volatile devices. In non-volatile devices, the resistance state is maintained after the electric field is removed. Representative examples include resistive random-access memory (RRAM), in which conductive filaments—typically composed of oxygen vacancies—remain thermodynamically stable [28,29], and ferroelectric memristors, in which the resistance state is determined by a non-volatile ferroelectric polarization [30]. In volatile devices, by contrast, the conductive pathways spontaneously decay and rupture upon bias removal, causing the device to revert to its high-resistance state (HRS). Volatile devices can be further classified according to their switching mechanisms. The first type is the diffusive memristor, which relies on active metal ions (e.g., Ag^+^ and Cu^+^) to form metallic filaments that spontaneously break upon bias removal due to interfacial energy minimization [31]. The second type is the Mott memristor, which exploits electrically induced insulator-to-metal phase transitions (e.g., in VO_2_ and NbO_2_ systems). The metal state in such devices is sustained by continuous Joule heating. Once the bias is removed, the material rapidly returns to its insulating state, and its I–V characteristic often exhibits a negative differential resistance (NDR) region [32]. Another volatile switching mechanism is represented by ovonic threshold switching (OTS) devices. OTS devices exhibit volatile threshold switching because electronic trap states in the amorphous chalcogenide are gradually filled with increasing voltage: below the threshold voltage carriers are trapped, resulting in HRS, whereas above the threshold voltage the traps become saturated and conductivity increases sharply, producing a low-resistance state (LRS) that spontaneously relaxes back to HRS after bias removal [33].

Since the first batch of memristors was successfully fabricated by HP Labs in 2008, these devices have demonstrated enormous potential in fields such as non-volatile memory, in-memory computing architectures and neuromorphic computing [34,35,36,37]. The advantages of simple structure, extremely low power consumption, fast read/write speeds and good compatibility with CMOS processes have contributed to this potential [38,39]. More recently, the diverse intrinsic random phenomena observed during memristor operation have established them as an ideal platform for implementing hardware security primitives [40,41,42]. Firstly, their resistive switching mechanism typically involves the random formation and breakage of conductive filaments within an insulating medium. Constrained by complex physical processes such as ion migration, thermal effects, and random nucleation, the process exhibits a high degree of intrinsic randomness [43,44]. Secondly, under specific resistance states, defect-induced random capture and emission of charge carriers generate significant random electrical noise, with the durations of the high and low levels acting as random variables that constitute a high-quality entropy source [45]. Through materials-science strategies such as deliberate electrode selection [46] and interface optimization [47,48], internal ion migration and filament stability can be precisely regulated, enabling a controllable transition from non-volatile to volatile switching behavior [49,50,51,52,53,54]. Moreover, structural designs including multi-layer heterostructures [47], crossbar arrays [55,56] afford precise control over the extent of conductive channel growth and disruption, yielding stable and reproducible multi-level resistive states [57,58,59,60]. These complementary attributes jointly provide a high-entropy, unpredictable physical source for constructing high-speed true random number generators, while simultaneously opening broader possibilities for the realization of more complex, higher-security encryption systems, such as physical unclonable functions and multi-valued logic hardware security modules.

In the development of hardware security primitives, the choice of material system for memristors directly governs the transition from a physical entropy source to a reliable security IP [61,62]. Compared with the inherent defects of organic materials in terms of thermal stability and environmental tolerance, as well as the significant process challenges associated with the wafer-scale uniform fabrication and CMOS back-end integration of two-dimensional materials, oxide-based memristors have become the predominant mainstream research platform in this field due to their outstanding comprehensive advantages [63]. Notably, transition metal oxides such as HfO_x_ and TaO_x_, themselves mature high-k dielectrics in advanced CMOS processes, can be seamlessly integrated into chips via standard back-end processing, thereby eliminating the risks of contamination and thermal budget mismatch that inevitably accompany the introduction of new materials [64]. Moreover, because their resistive switching mechanism relies on the formation and fracture of oxygen-vacancy-conductive filaments, these devices deliver extremely high endurance and excellent data retention, ensuring that the ‘fingerprint’ can be consistently reproduced with a low bit error rate throughout the entire lifecycle of a secure chip [65]. Crucially, physical constraints enable precise regulation of the operating conditions, thereby allowing controllable extraction of high-quality entropy through precise regulation of operating voltage and current limiting, whilst simultaneously avoiding the detrimental effects of uncontrollable fluctuations on reliability. Indeed, it is precisely this optimal balance between process compatibility, device reliability, freedom to control randomness, and the capability for high-density cross-array integration that has established the dominant position of oxide memristors in current hardware security research [66].

While previous reviews laid an important foundation for understanding memristor-based hardware security research, their coverage and classification depth still exhibited certain limitations. Pang et al. systematically summarized the principal design methodologies for PUFs and TRNGs, yet the works they examined were primarily confined to 2018 and earlier [67]. Lv et al. extended the discussion into the domain of chaotic circuits, but their classification of TRNGs remained relatively cursory and failed to adequately reflect the performance disparities among different entropy source types [40]. Furthermore, none of the aforementioned reviews provided a comprehensive performance table, which restricted the readers’ ability to compare different schemes. To remedy these deficiencies, this work updated the literature from recent years, provided a systematic classification and exposition of TRNGs and PUFs, and compiled a detailed performance summary table organized by key metrics, as presented in Table 1. Meanwhile, we also discussed other memristor-based hardware security solutions that had attracted less attention in previous reviews and further expanded the discussion at the application level. Together, these efforts constituted the primary advancement of the present review relative to existing studies.

**Figure 2:**
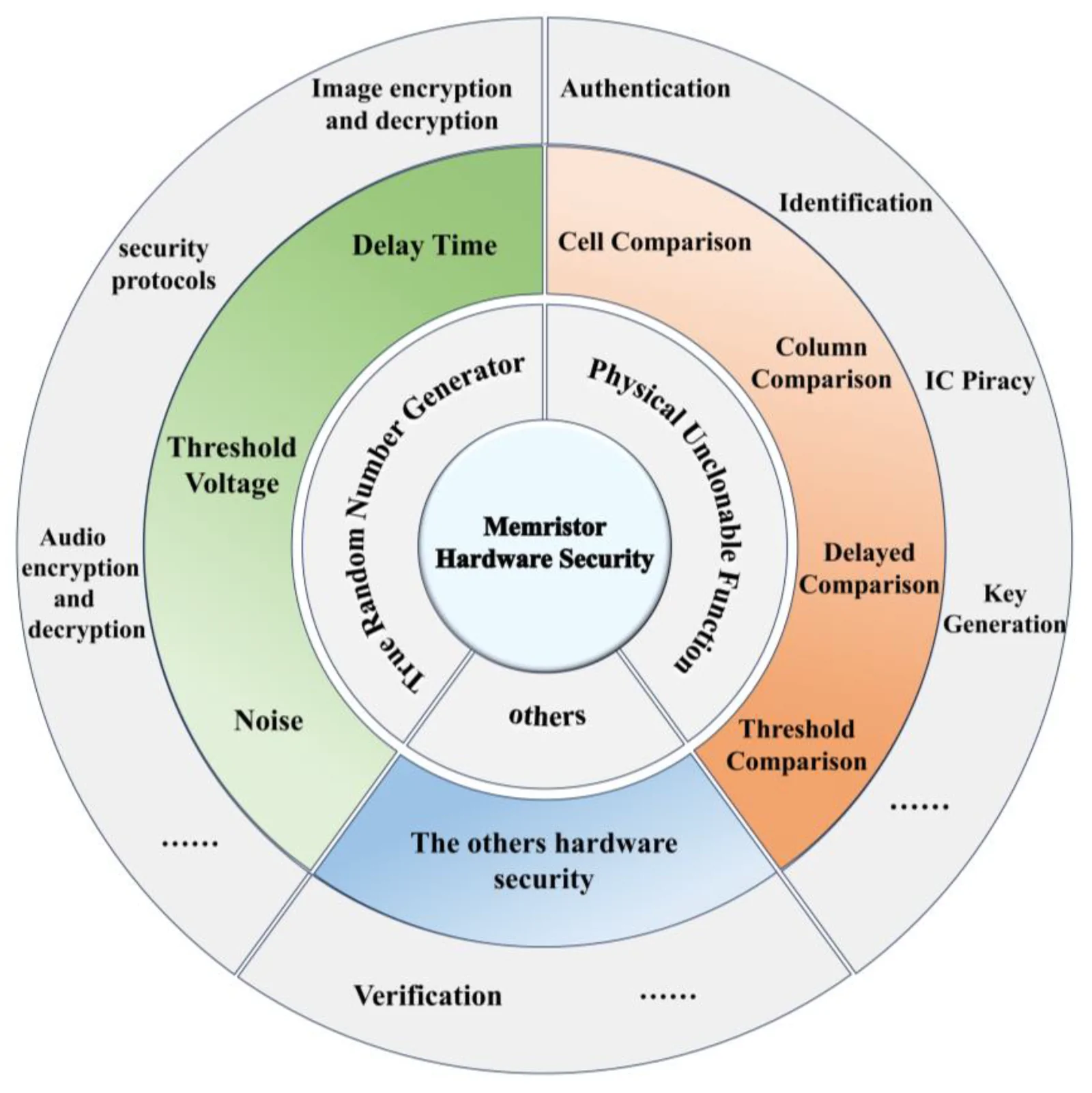
Schematic diagram of hardware security solution based on memristors.

The specific details are shown in Figure 2, where we provide an overview of TRNG implementation schemes categorized by entropy source, focusing on their respective throughput, power consumption, and random number quality. We then classify PUFs by bit-generation strategy for the first time [40,67], dividing them into four categories: cell comparison, column comparison, delay comparison and threshold comparison. Furthermore, it systematically compiles a comparative table of key performance metrics for TRNGs and PUFs, providing a clear engineering data sheet to aid in the selection of appropriate solutions. Furthermore, in contrast to other reviews, this work introduces other, less-explored memristor-based hardware security solutions, such as non-volatile secure storage, logic obfuscation circuits, and physical attack detection circuits, demonstrating their potential in information hiding, secure transmission, and authentication. Additionally, the manuscript significantly broadens the scope of applications, extending from simple key generation to system-level scenarios such as image/voice encryption, secure protocols, chip anti-counterfeiting, and even homomorphic encryption acceleration, revealing the full-chain evolutionary trend of memristor security technology moving from key generation to key utilization and key protection. Most importantly, these findings provide profound insights into the core bottlenecks hindering industrial implementation, identify the primary obstacles to practical deployment, and propose future research directions at the device, circuit and system levels, raising the discussion from basic laboratory research to the strategic level of engineering deployment. Collectively, we present a ‘roadmap’ for the design of next-generation, highly secure, low-power hardware security chips that combines theoretical depth with practical guidance.

The literature for this review was collected through a multi-database, iterative search process rather than a single search based on a fixed search string. Given that this review covers several related but distinct areas, including TRNGs, PUFs, and other memristor-based hardware security approaches, multiple rounds of literature searches were conducted throughout the preparation and revision of the manuscript. Relevant studies were primarily identified from IEEE Xplore, Web of Science, and Google Scholar using combinations of memristor-related terms, such as “memristor”, “memristive device”, “RRAM”, “ReRAM”, and “resistive switching”, together with hardware-security-related terms, including “true random number generator”, “TRNG”, “physical unclonable function”, “PUF”, “hardware security”, and “cryptographic hardware”. Candidate publications were evaluated based on their relevance to the scope of this review. Particular attention was given to studies in which memristive or resistive-switching devices played a direct role in hardware security functions, including random number generation, physical unclonability, encryption, authentication, secure storage, and related security mechanisms. Studies focusing exclusively on conventional memory, neuromorphic computing, or in-memory computing without a direct hardware security function, as well as publications in which memristors were mentioned only as background technologies, were not included in the core analysis. Relevant publications were further supplemented through manual examination of previous reviews, key references, and backward and forward citation tracking. Therefore, the final bibliography represents the outcome of multiple rounds of database searches, manual evaluation, and citation tracking, and includes both core studies directly related to memristor-based hardware security and supporting references providing relevant technical and scientific context.

## 2. Design and Application

### 2.1. True Random Number Generator Based on Memristors

True Random Number Generator (TRNG) is a core component in hardware security systems, deriving its randomness from unpredictable physical processes. The randomness within memristors fundamentally originates from the coupled fluctuations of multi-physical processes at the nanoscale, forming a multi-level stochastic system that provides a natural entropy source for TRNG construction. Specifically, the static disorder in the spatial distribution of point defects such as oxygen vacancies within the functional dielectric, coupled with their dynamic random hopping under the influence of electric fields and thermal excitation, continuously generates random electrical noise in the local conductance. Concurrently, charge carrier migration driven by electric fields exhibits unpredictable path selection and step size distributions within inhomogeneous grain boundaries and amorphous networks, thereby rendering the morphological evolution of conductive channels highly uncertain. Building upon this, the nucleation sites of conductive filaments are determined by local defects and the regions of strongest electric field, whilst the extent of Joule heating dissolution and the size of the breakdown gap during their fracture are entirely random, enabling the switching between HRS and LRS to directly generate random variables available for sampling. Incorporated within the operating mechanism of memristors themselves, these cross-scale stochastic processes, ranging from atomic-level defect transitions to device-level resistance switching, enable the generation of compact, low-power-consumption, process-compatible high-speed true random numbers without the need for additional high-entropy source circuits. As mentioned above, the required randomness is dynamic and time-domain in nature. The entropy source must exhibit significant cycle-to-cycle (C2C) fluctuations in parameters such as switching delay time, threshold voltage, relaxation time, or read current noise [67]. Each switching event should be as independent and unpredictable as possible. High entropy, high throughput, and the ability to pass statistical randomness tests are the primary performance metrics. In this context, device variability is not a source of interference, but rather a ‘resource’ to be harnessed. Depending on the source of entropy, existing memristor-based TRNGs can be broadly categorized into these three categories: delay-time TRNG, threshold voltage TRNG, and noise-based TRNG.

#### 2.1.1. Delay Time TRNG

In memristors, the interval between the application of an input signal and the resulting transition from the HRS to the LRS is defined as the delay time, while for volatile memristors the interval required for the device to spontaneously relax back to the HRS after the removal of the voltage input is termed the relaxation time. The extraction of parameters based on the delay time and relaxation time is a key approach to the implementation of memristor-based TRNGs.

**Figure 3:**
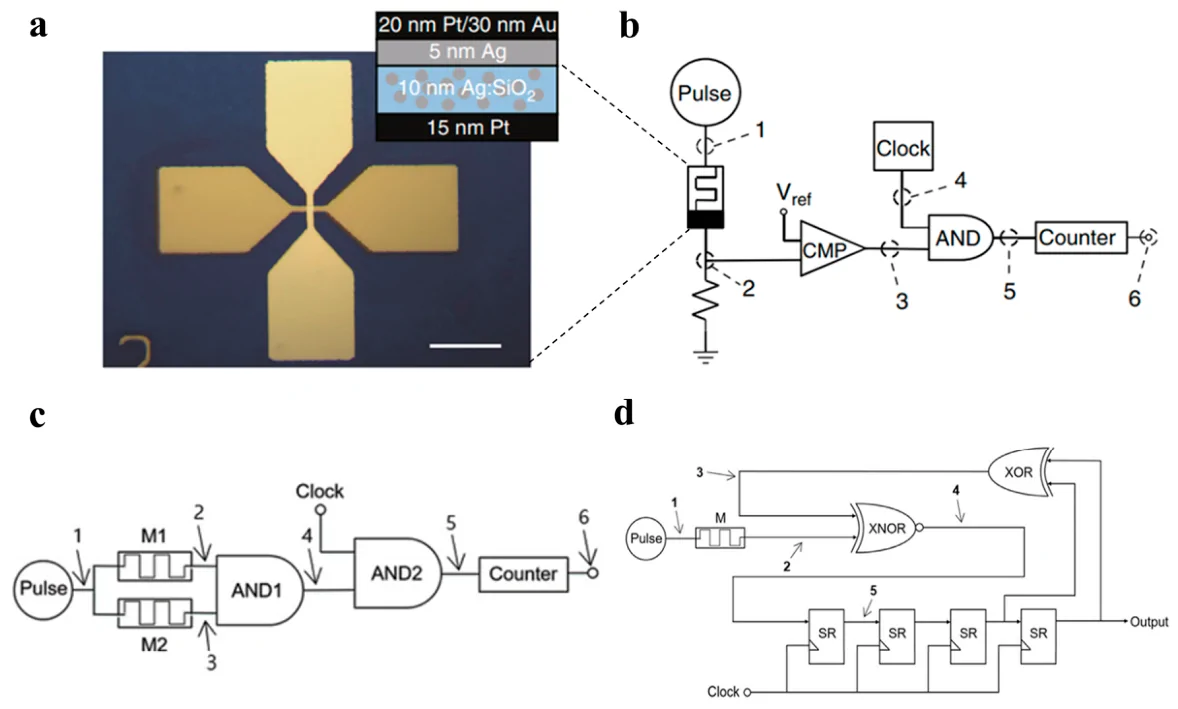
True random number generators (TRNGs) with delay time as an entropy source. (**a**) Optical micrograph of a 5 × 5 µm^2^ Ag: SiO_2_ junction device. Scale bar, 50 µm. The inset illustrates the stacked structure of the Ag: SiO_2_ diffusive memristor [68]. (**b**) TRNG circuit diagram based on an Ag: SiO_2_ diffusive memristor [68]. (**c**) Schematic diagram of a dual-channel TRNG based on Pt/HfO_2_/TiN memristors [69]. (**d**) TRNG schematic diagram based on a Pt/HfO_2_/TiN memristor incorporating an NFSR [70].

In early research on volatile memristors, Jiang et al. fabricated a TRNG in 2017based on an Ag: SiO_2_ diffusive memristor, with its device structure and circuit design shown in Figure 3a and Figure 3b, respectively. The circuit connects the memristor in series with a resistor for voltage division and employs a combination of clock and counter circuits. When a high-level electrical pulse is applied, the memristor enters LRS after a random delay time, during which the counter continuously increments, and the parity of the final count determines the output bit (0 or 1). This work marked the first implementation of the volatile memristor-based TRNG capable of passing NIST testing without post-processing. However, it did not utilize the characteristic of relaxation time inherent in volatile memristors [68]. To address this, in 2018, Woo et al. from Hwang’s team proposed a TRNG design based on a Pt/HfO_2_/TiN memristor that innovatively employs random relaxation time as the second entropy source, as shown in Figure 3c. By applying the input signal to both memristors simultaneously, the widths of the outputs V2 and V3 are governed by their respective delay times and relaxation times, and the random output is generated from the overlapping region of these dual random pulse widths, which not only enriches the entropy sources but also improves circuit lifetime [69]. Subsequently, as shown in Figure 3d, they innovatively introduced a non-linear feedback shift register (NFSR) into the memristor TRNG design, injecting physical randomness into each iteration of the nonlinear feedback network to significantly enhance the unpredictability of the output bit sequence, although the maximum bit rate remained limited to 32 kb/s [70,71]. Nevertheless, the introduction of NFSR has provided new avenues of exploration for subsequent researchers. For example, the TRNG designed by Yan et al. utilizes Ag/SiN_x_/n-Si memristor devices with delay and relaxation times in the nanosecond range. Furthermore, while separating a single clock signal, it utilizes both the rising and falling edges to drive two independent yet synergistic NFSRs, thereby achieving a bit output rate of 112 kb/s for the TRNG [72]. The performance of the memristors described above is shown in Table 2.

Nonvolatile memristor TRNGs typically rely on a single-trigger mode, in which random delay times generate random pulse widths and the number of pulses within that interval is counted to produce random bits, which, for the first time, achieved an on-time of approximately 30 ns and an off-time of 20 ns on an amorphous silicon substrate, substantially outperforming other threshold switching devices [73]. In a fundamentally different approach, Gu et al. designed a TRNG based on a TiN/NbO_x_/Pt Mott memristor that operates in continuous self-oscillation mode, exploiting the random delay times arising from the Joule-heating-induced Mott phase transition to generate sustained random oscillations via a 1T1R circuit. After the oscillation waveform is shaped into a square wave by a comparator, a D flip-flop samples the output at the rising edge of the clock. Notably, the oscillation frequency can be tuned by adjusting the transistor gate voltage, allowing the system to dynamically scale throughput—reducing frequency for power saving and increasing it for high-speed encryption—thereby significantly enhancing environmental adaptability and demonstrating the potential of rate tunability to balance power consumption and speed [74]. In a noteworthy development, Wang et al. proposed a flexible memristor-based TRNG in 2025, utilizing Ti_3_C_2_ MXene-doped polyethyleneimine (PEI), which achieves dual-mode switching through precise control of the doping concentration. Below a doping ratio of 300:1, it exhibits non-volatility, whilst above a doping ratio of 600:1, it exhibits volatility. This represents the first integration of these two functions within a single material system [75]. Although the necessity of waiting for the memristor to switch states means that the delay time imposes a lower limit on the output rate of TRNG bits, this approach delivers excellent resilience to environmental drift and generates extremely high intrinsic randomness without post-processing. Consequently, among the three TRNG types discussed in this section, delay-time-based designs offer high intrinsic randomness quality with minimal post-processing requirements, making them particularly suitable for lightweight IoT security applications where environmental robustness is prioritized over throughput.

#### 2.1.2. Threshold Voltage TRNG

The resistive switching behavior in memristors originates fundamentally from the nanoscale formation and rupture of conductive filaments, a process synergistically governed by electrically driven ion migration, redox reactions, and localized Joule heating. Crucially, every step of ion movement is subject to uncontrollable factors, including migration paths, reaction rates, and thermal field distributions, all of which are profoundly influenced by thermal fluctuations, inhomogeneities in defect distribution, the degree of local electric field concentration, and compositional variations at the atomic scale. As a result, significant random fluctuations are inherent to both the forward progression from filament nucleation and growth through to electrode connection and the subsequent Joule-heating-dominated dissolution and fracture stages, permeating the microscopic evolution and transient structures alike. When this intrinsic randomness t is transduced to the macroscopic electrical response, the threshold voltage no longer behaves as a deterministic constant but instead follows distinct statistical distribution characteristics, typically described by Gaussian, log-normal, or Weibull statistics. TRNGs that utilize the random fluctuations in threshold voltage as their core entropy source are referred to as threshold voltage TRNGs.

**Figure 4:**
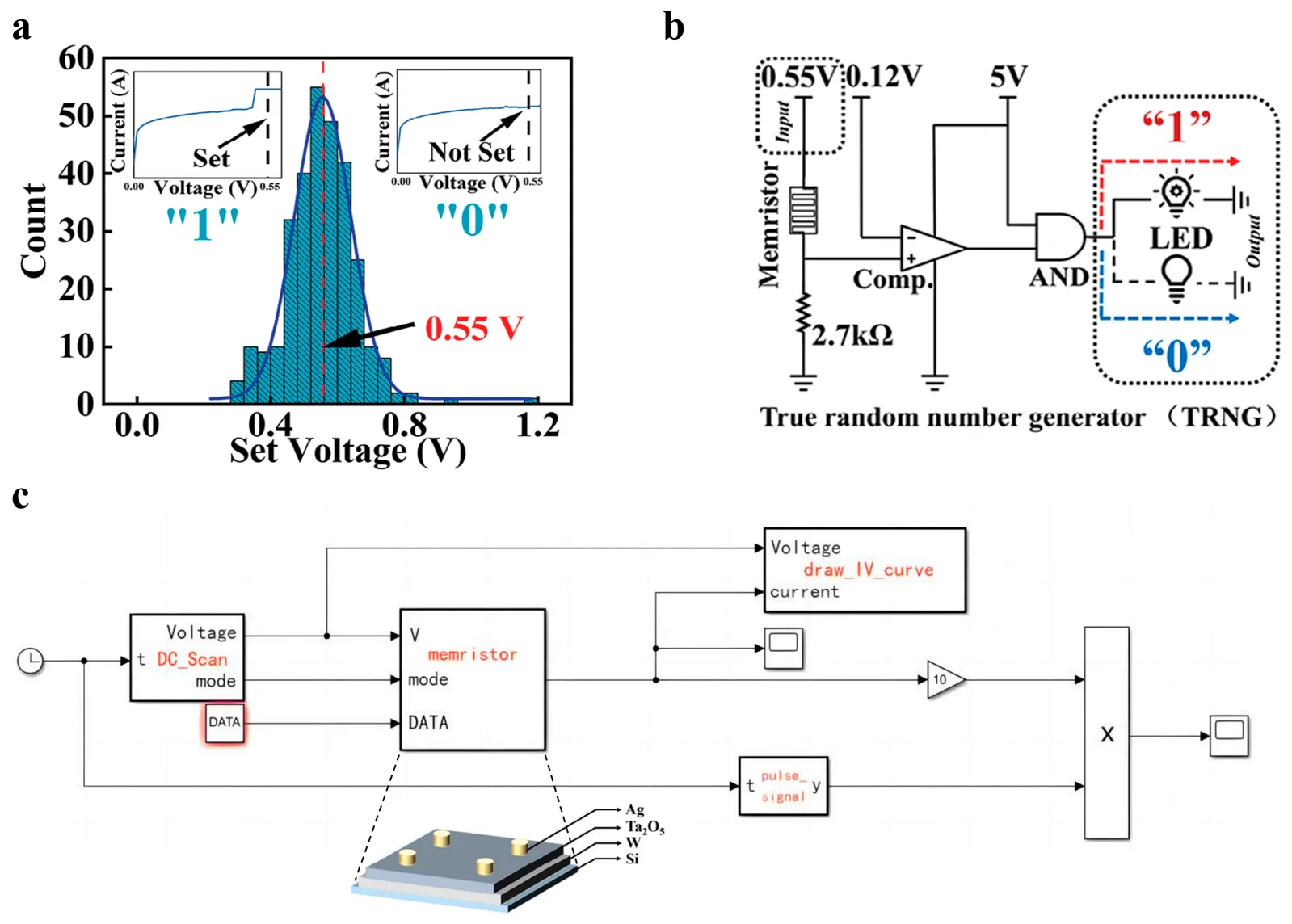
True random number generators (TRNGs) using threshold voltage as an entropy source. (**a**) Set voltage distribution for 300 cycles [76]. (**b**) TRNG circuit diagram based on a Ta/BiFeO_3_/ZrO_2_/Pt bilayer memristor [76]. (**c**) TRNG circuit diagram based on an Ag/Ta_2_O_5_/W memristor. The inset illustrates the stacked structure of the memristor [80].

In memristor-based TRNGs, the cycle-to-cycle variability of the set voltage (V_SET_) and reset voltage (V_RESET_) constitutes a primary physical entropy source, giving rise to two mainstream design strategies. The first, as illustrated in Figure 4a, directly applies the statistically determined median V_SET_ to the memristor in its HRS. At this point, the memristor theoretically has a probability of nearly 50% of switching to the LRS, allowing the bit to be output by directly reading the state of the memristor. Alternatively, as shown in Figure 4b, a comparator combined with an AND gate circuit is used to process the voltage divider produced by the memristor connected in series with a resistor, the voltage magnitude of which is determined by the state of the memristor. If the memristor switches to the LRS, resulting in a higher voltage across the resistor, it outputs ‘1’, whereas the device remaining in HRS produces a lower voltage and outputs ‘0’ [76]. However, this approach does not efficiently exploit the available entropy source. To improve efficiency, Yang et al. discovered that the Pt/Ti/TaO_x_/Pt memristor exhibits significant voltage fluctuations during both the SET and RESET processes. Consequently, they extract the median values of both V_SET_ and V_RESET_, sequentially outputting two random bits within a single operation cycle. Although this doubles the per-cycle bit generation relative to conventional single-bit schemes, the requisite conditional branching introduces substantial timing overhead, thereby constraining practical throughput [77]. To further improve the actual bit output rate, the TRNG designed by Cheng et al. achieved high throughput of 5 Mb/s and low power consumption of 1.8 nJ/bit, as shown in Table 2. The VO_2_/HfO_2_ stacked dual-mode memristor exhibits nanosecond-scale volatility and relaxation time, eliminating the reset steps required by traditional nonvolatile devices and enabling continuous high-speed pulse inputs. Such devices are exceptionally suited for applications requiring high-speed data streams, like random number generation [78]. Beyond efficiency, multifunctional integration remains the ultimate research objective. For instance, the Ag/PVP-MQDs/Si photoelectric memristor TRNG reported by Huang et al. in 2025 achieved dual integration of neuronal and TRNG functions under the synergistic control of light and electricity, demonstrating flexible switching and rapid response [79]. Furthermore, the ZrO_2_/BiFeO_3_-based memristor TRNG designed by our team can also achieve functional integration by adjusting the current limit. Although switching flexibility is limited, it delivers favorable performance metrics and strong CMOS compatibility, facilitating large-scale array integration [76].

Another design approach, illustrated in Figure 4c, is embodied by the Ag/Ta_2_O_5_/W structure memristor TRNG designed by Wang et al. [80]. In this scheme, a scanning voltage is ramped from zero, and the median value of V_SET_ is adopted as the threshold, such that a SET event occurring before the scan reaches this median yields a logical ‘1’, whereas the absence of such an event yields a logical ‘0’. Additionally, the Al/AlO_x_/Bi_2_O_2_Se/Pd memristor TRNG [81] and the Al/AlO_x_/graphene memristor TRNG [82], both proposed by Liu et al., also employ the same design approach. It is worth noting that both of these TRNGs feature dual modes: digital and analogue. In the analogue mode, the entropy source is noise, which will be explained in the next section. The latter also incorporates a PUF function, which will be discussed in the following chapter. Although TRNGs utilizing threshold voltage as an entropy source may suffer from aging-induced drift and sensitivity to environmental fluctuations as usage accumulates, they also offer advantages such as simple circuitry, rapid response, controllable power consumption, and compatibility with CMOS processes. Looking ahead, advances in two-dimensional material interface engineering, including the atomic-level precise control of oxygen vacancy migration barriers, combined with circuit systems, will work synergistically to mitigate aging effects and enhance environmental stability. Thereafter, the TRNGs of this type will be capable of combining high speed, long lifespan, and resilience against physical attacks, and are expected to play an increasingly important role in fields such as hardware security, random computing and IoT encryption. Relative to delay-time-based designs, threshold-voltage TRNGs achieve substantially higher throughput at the cost of increased sensitivity to environmental fluctuations and aging effects.

#### 2.1.3. Noise-Based TRNG

Because intrinsic noise in conventional CMOS circuits is typically extremely faint, its extraction requires a low-noise, high-gain amplification chain, and this process is highly susceptible to coupling with power supply ripple, substrate interference and electromagnetic crosstalk, which degrades the quality of the entropy source while imposing persistently high power consumption and circuit complexity. In contrast, memristors exhibit rich and significant current fluctuations at the nanoscale, originating from non-equilibrium physical processes such as oxygen vacancy migration and the random breakage and reconnection of conductive filaments. The inherent unpredictability of these fluctuations makes noise-based TRNG one of the most direct and extensively investigated approaches for memristor-based security applications, where the efficient and reliable conversion of random fluctuating analogue signals into a high-throughput digital bit stream essentially determines the ultimate performance of the entire TRNG. Since different memristor systems exhibit significant differences in statistical characteristics such as the distribution of switching amplitude, average residence time and power spectral density, and since design objectives vary in the trade-offs among random throughput, power consumption, area and resilience to environmental attacks, the circuit architectures of current noise-based memristor TRNGs are highly diverse.

**Figure 5:**
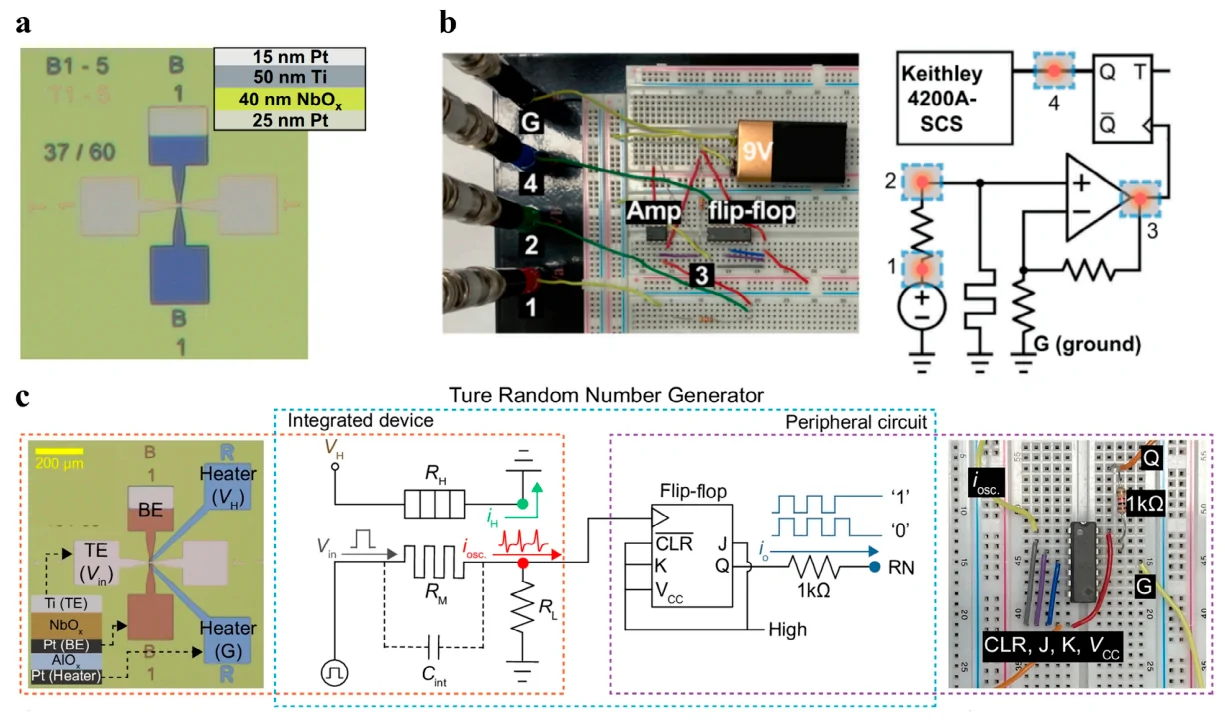
True random number generators (TRNGs) utilizing noise as an entropy source. (**a**) Optical microscope image of a Pt/Ti/NbO_x_/Ti/Pt memristor. The inset shows its stacked structure [83]. (**b**) Photograph of a TRNG circuit based on a Pt/Ti/NbO_x_/Ti/Pt memristor assembled on a breadboard (**left**) and the corresponding TRNG circuit layout (**right**) [83]. (**c**) The overall optimized system configuration, including the electrical circuit layout and the optical microscopy image of the components of the proposed TRNG. The integrated device was loaded into the probe station, while the T flip-flop was built on the breadboard [84].

Whenever the use of noise as a source of entropy is discussed, the conversation inevitably turns to Mott memristors. Near the critical point of the Mott phase transition, the device is extremely sensitive to even the slightest disturbance, generating significant noise. Thus, Mott memristors are ideally suited for utilizing noise as the source of entropy. As a representative class of Mott memristors, NbOx-based devices were employed by Kim’s team, who fabricated a Pt/Ti/NbOx/Ti/Pt structure as shown in Figure 5a, and under constant voltage bias, this device spontaneously produces current oscillations whose period is slightly modulated by thermal noise, thereby yielding random parity in the number of oscillation pulses. Building upon this behavior, they designed a self-clocked TRNG, as shown in Figure 5b, and implemented it on a circuit board. The core mechanism involves the NbO_x_ Mott memristor connected in series with a load resistor RL to generate self-oscillation. The voltage amplitude is then amplified by an inverting amplifier to a level sufficient to trigger the subsequent T-trigger, and the oscillation parity is converted into bits by the negative-edge-triggered T-trigger [83]. To enhance the bit output rate, as shown in Figure 5c, they subsequently integrated a resistive heater beneath the NbO_x_ memristor, actively raising the device temperature through physical means to enhance thermal noise and thereby increasing the throughput rate from 40 kbit/s to 100 kbit/s, a result that demonstrated the feasibility of actively regulating noise entropy sources through thermal engineering and opened new avenues for TRNG design based on thermal noise [84]. In fact, as can be seen from Table 2, the entropy source for the highest-output-rate memristor-based TRNGs is noise, exemplified by the TiN/TaO_x_/HfO_x_/TiN memristor TRNG [85] and the TiN/OGL/MO/HfO_x_/TiN memristor TRNG [86], both designed by Wu’s team, which are designed by utilizing the randomness in capacitance charging times resulting from noise fluctuations in the readout current of memristors. Although the former does not specify the throughput rate directly, the TRNG clock cycle is merely 5 ns, implying that a single-pass throughput on the order of Gbps is theoretically achievable, whereas the latter charges the capacitor via a 2T2R cell and determines the bit output by the margin between the capacitor charging time and the period of a three-stage ring oscillator, achieving an impressive throughput rate of 41.7 Mbps when implemented in a 28-nanometer process. The good uniformity and excellent throughput rate achieved in these demonstrations underscore the immense potential of thermal noise as an entropy source in practical chip-level integration.

**Figure 6:**
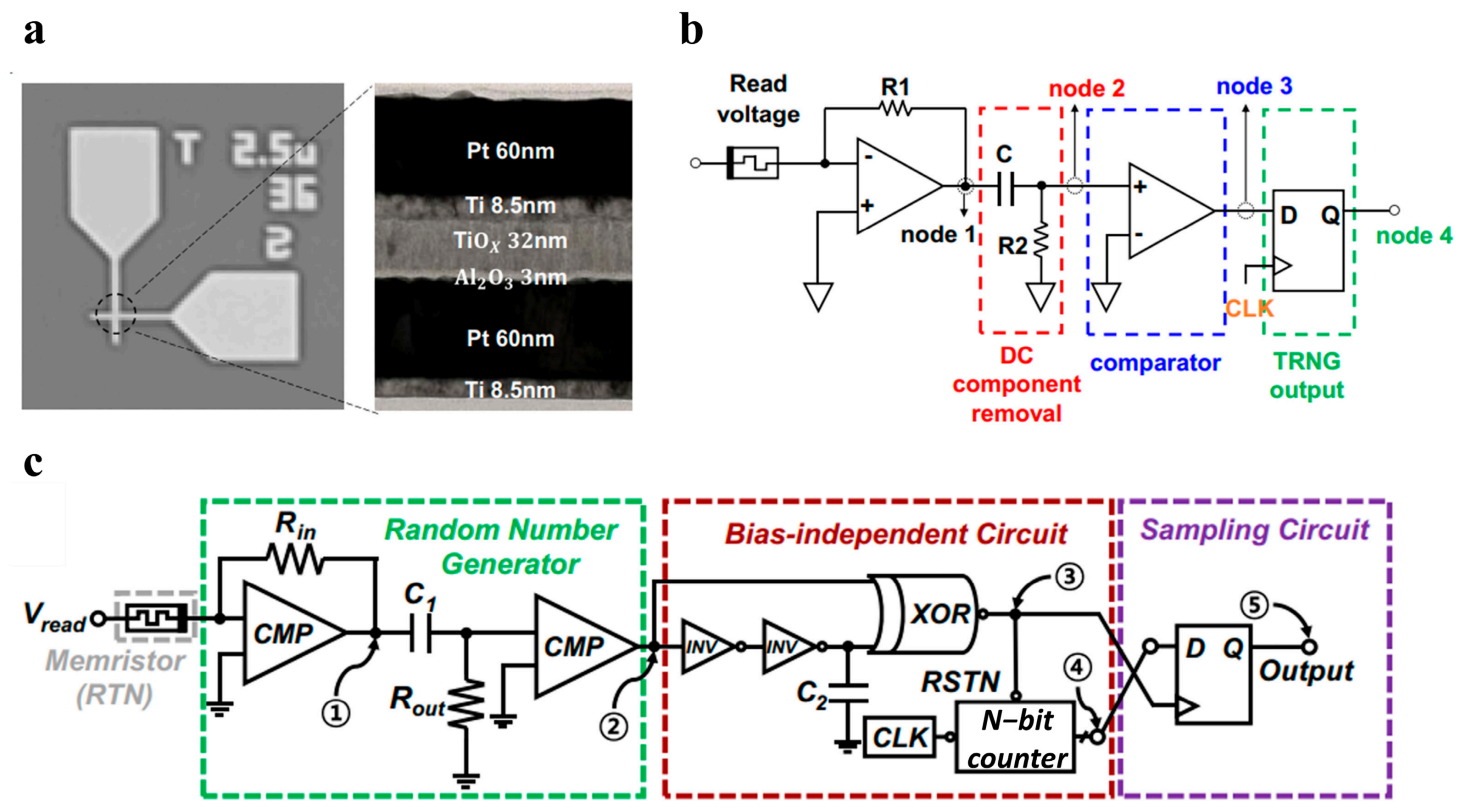
True random number generators (TRNGs) utilizing Random Telegraph Noise as an entropy source. (**a**) Top view schematic and cross-sectional transmission electron microscopy (TEM) image of the Pt/Ti/TiO_x_/Al_2_O_3_/Pt/Ti memristor device [87]. (**b**) Schematic diagram of the TRNG circuit based on the memristor shown in (**a**) [87]. (**c**) Schematic diagram of the optimized TRNG circuit [88].

Random Telegraph Noise (RTN), a type of noise in semiconductor devices characterized by random jumps in current or conductance between discrete levels, arises from the random capture and release of electrons by defect energy levels. When a trap captures an electron, the conduction channel becomes further depleted and the current jumps to a low-level state corresponding to the capture time τ_c_, whereas the release of the electron restores the high-level state corresponding to the emission time τ_e_. The random nature of this physical process makes RTN the core source of entropy in noise-based memristor TRNGs. Generally, a uniform random bit stream can be obtained by directly sampling the RTN signal, as demonstrated by the Pt/Ti/TiO_x_/Al_2_O_3_/Pt/Ti memristor designed by Kim’s team and shown in Figure 6a, the TRNG circuit design illustrated in Figure 6b samples, amplifies, DC-blocks and compares the RTN signal before the bit stream is directly output by a D-flip-flop [87]. However, because τ_c_ and τ_e_ vary exponentially with read voltage and temperature, direct sampling can lead to severe bit bias. To address this issue, they adjusted the thickness of the intermediate layer and further optimized the circuit to propose a bias-independent TRNG architecture, as shown in Figure 6c, in which an edge detection circuit identifies the transition edges of the RTN signal and utilizes these edges as reset signals for an N-bit counter, such that the output value of the counter is determined by its state at the moment of the transition and the random transitions of the RTN are converted into a random number output. In this case, the randomness of the output bits derives from the timing of the transition event rather than the level of the signal, which fundamentally eliminates the influence of the τ_c_/τ_e_ ratio bias [80]. As mentioned in the previous section, the Al/AlO_x_/Bi_2_O_2_Se/Pd memristor TRNG [81] and the Al/AlO_x_/graphene memristor TRNG [82], proposed successively by Liu et al., operate in dual modes with RTN serving as the entropy source in the analogue mode. The former does not convert the RTN signal into a digital bit stream but instead uses it directly as an analogue key for the encryption and decryption of voice signals, thereby overcoming the limitation of traditional TRNGs that generate only digital bit streams and providing a direct security solution for processing continuous analogue signals such as biometric and environmental sensing data. Although the latter directly samples the RTN signal in analogue mode, the work innovatively combines the two modes and introduces Fibonacci-type linear feedback shift register technology, which, based on a 16-bit initial seed, can generate 2^16^ − 1 = 65,535 random numbers per cycle and offers a new approach to enhancing random number generation rates. TRNG designs that utilize noise as an entropy source each have their own specific focus, creating a wide range of design options in terms of throughput, power consumption, area, resistance to bias, and post-processing complexity, thereby offering diverse choices for optimizing TRNGs across different application scenarios. Among the three TRNG types, noise-based designs deliver the highest throughput, but require the most sophisticated readout circuitry and are most susceptible to external interference, so the choice among the three approaches ultimately depends on the specific application priorities regarding speed, power, area, and environmental robustness.

In general, memristor-based TRNGs exhibit significant differences across entropy source types, which have profound implications for practical deployment. Delay-time TRNGs can still pass NIST tests at elevated temperatures, and some devices fabricated on flexible substrates maintain stable resistive switching characteristics under a bending radius of 1 mm and after 2000 bending cycles [71,75]. In contrast, the median threshold voltage in threshold-voltage TRNGs undergoes systematic shifts with increasing temperature, read voltage, and cycling counts, severely degrading randomness quality and exhibiting poor environmental robustness. Among noise-based TRNGs, those using thermal noise as the entropy source benefit from moderately elevated temperatures that facilitate entropy generation [84], whereas those relying on random telegraph noise (RTN) are sensitive to temperature-induced changes in capture and emission time constants [88]. For RTN-based designs, bias-insensitive circuit architectures that decouple the output from the time constant ratio are required to maintain stable randomness.

Nevertheless, it should be noted that systematic quantitative comparisons of supply-voltage variation, read-voltage variation, humidity, mechanical deformation, and aging remain largely absent from the existing memristor-based TRNG literature. Most reported works have focused primarily on randomness quality, throughput, and basic reliability metrics such as retention and endurance, whereas comprehensive environmental robustness has not yet been systematically evaluated. The above discussion is therefore based on the limited data currently available, and a dedicated quantitative assessment of these environmental factors across different TRNG categories represents an important direction for future research.

#### 2.1.4. Applications of TRNG Based on Memristor

**Figure 7:**
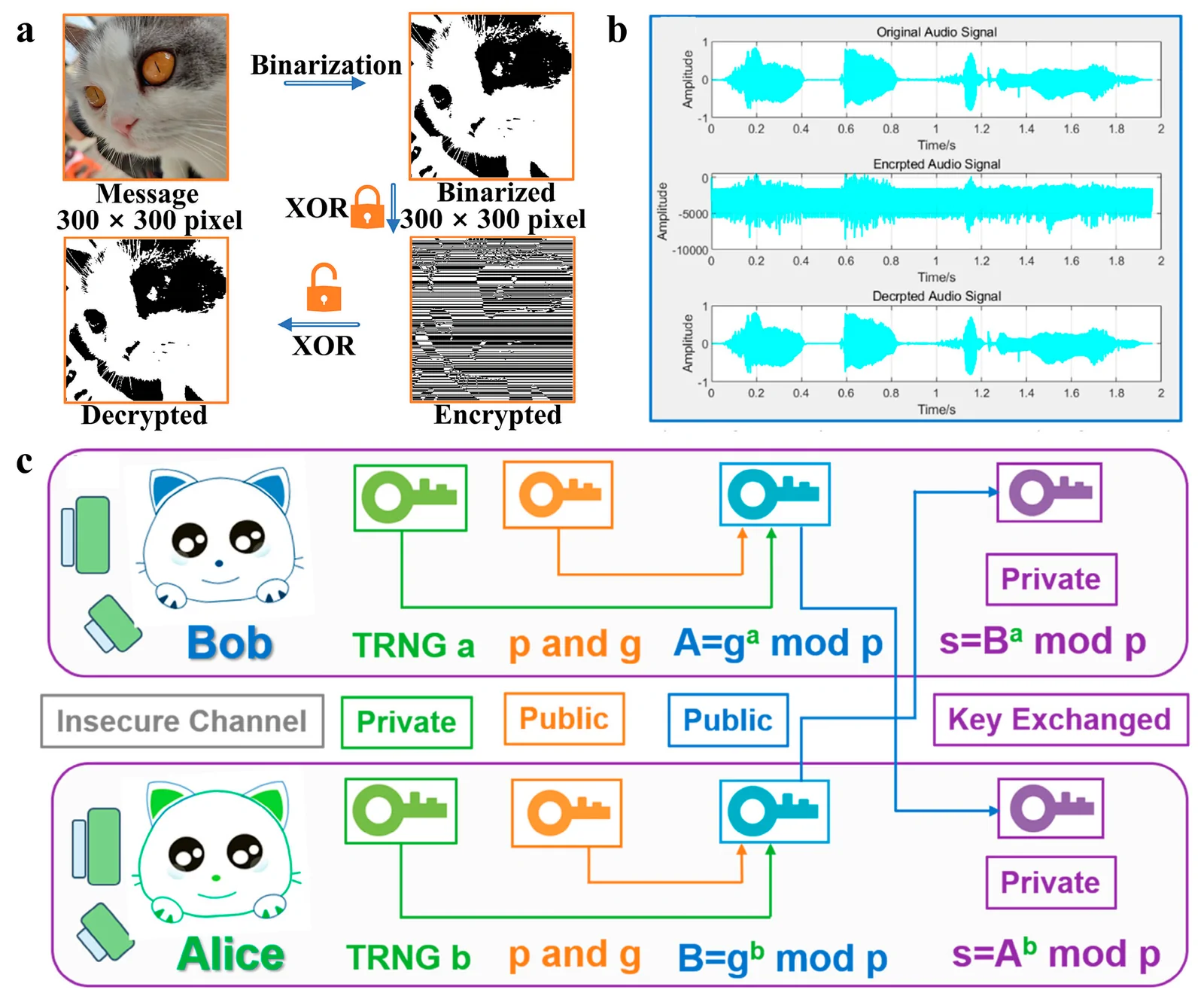
Applications of True random number generators (TRNG) based on memristor. (**a**) The process diagram of image encryption and decryption is based on image binarization and XOR logic [76]. (**b**) Original, encrypted, and decrypted female audio signal of “Hi, Bob. Happy New Year” [81]. (**c**) Illustration of TRNG-based Diffie–Hellman Key Exchange protocol, where Bob and Alice successfully realize key exchange through an insecure channel [81].

Research on memristor-based TRNGs is currently evolving from single key generation functions towards integrated hardware security systems that combine high security, high energy efficiency, and intelligence. Building on this, various studies have utilized different material systems and circuit designs to achieve diverse applications of TRNGs in data encryption, in which TRNG-generated keys are combined with plaintext through XOR operations to achieve lossless encryption and decryption of MNIST handwritten digits as well as binary and grayscale images, and the significant reduction in correlation coefficients between adjacent pixels of the encrypted images validates the reliability of the hardware keys [43,76,80]. To illustrate the specific details, the encryption and decryption of a grey cat image by the TRNG designed by our team is taken as an example. Figure 7a illustrates an image encryption and decryption system based on a ZrO_2_/BiFeO_3_ bilayer memristor. In the original study, the software simulation of image encryption used 300 random numbers generated by the TRNG as the key and then repeated these 300 random numbers 300 times to form a 300 × 300-pixel key image. It should be noted that repeating the same key stream introduces strong periodic structures, which is cryptographically insecure. Generating 90,000 random numbers to construct a 300 × 300-pixel key image is a recommended improvement. In this improved scheme, first, a software algorithm converts an image of a cat into a 300 × 300-pixel binary image, where black corresponds to ‘1’ and white to ‘0’. Subsequently, the TRNG generates 90,000 random numbers to construct a 300 × 300-pixel key image, and the generated key bits are combined with the binary image information via an XOR operation to accomplish encryption. The simulation results demonstrate the functional feasibility of the encryption approach. Finally, the encrypted image is restored to its original form by applying the XOR operation again with the previously generated key image, thereby completing the entire encryption and decryption process. Software simulation has verified the feasibility of combining image encryption with TRNG key generation, whereby the hardware-generated key demonstrates the functional feasibility of the encryption approach. Furthermore, research trends are no longer confined to isolated data encryption and decryption. Instead, by utilizing the controllable volatile and nonvolatile behavior of memristors within the same device, image recognition and encryption functions are integrated into a single array. Employing a ‘selective encryption’ strategy to protect only critical information, the efficiency of information transmission is increased threefold, achieving deep integration of computational and security capabilities [79].

In addition to image encryption and decryption, memristor-based TRNGs can also be used for audio encryption and decryption, as well as secure protocols. Of particular note is the dual-mode TRNG developed by Liu et al. based on Bi_2_O_2_Se memristors, which is capable of performing both of these functions simultaneously. On the one hand, it utilizes the analogue fluctuations of the RTN signal as an analogue entropy source, generating RTN signals with five distinct amplitude characteristics by adjusting the back-gate voltage. Using a symmetric-key algorithm, the five RTN signals are combined to form an encryption key, which is then used to obfuscate and encrypt the voice signal. As shown in Figure 7b, the encrypted voice is completely unrecognizable to the human ear, whereas the original voice is restored without loss upon decryption. In contrast, the simple XOR encryption method can only conceal approximately 50% of the information, and the human ear can still recognize the original sound. This marks the first instance of audio encryption in a memristor TRNG application. However, if an eavesdropper intercepts the encrypted speech and discovers the algorithm, communication security is compromised. Therefore, a secure transmission protocol must be introduced to protect the key distribution process.

Thus, on the other hand, utilizing the bit stream generated by the TRNG, the Diffie–Hellman key exchange protocol is implemented to ensure the security of communications over an insecure channel. Based on the generated random numerical keys, Figure 7c illustrates the Diffie–Hellman key exchange protocol using the TRNG [81]. The protocol begins with the two communicating parties (Bob and Alice) each using the TRNG to generate private random integers a and b. Then, the parties exchange their respective public keys via an insecure channel. Finally, each party calculates (g^b^)^a^ mod p = (g^a^)^b^ mod p to derive the shared private key. The Diffie–Hellman key exchange protocol was originally proposed by Diffie and Hellman in 1976, with the aim of jointly establishing shared secret information via public keys and random numbers over an insecure channel. The security of the algorithm is based on the difficulty of solving the discrete logarithm problem. Suppose an attacker attempts to break this shared secret. He would need to compute either (B^a^ mod p) or (A^b^ mod p). As he does not know the random integers a or b, he cannot derive the secret key. However, if the attacker knows either a or b, he could compromise the security of the system, which raises the issue of forward secrecy (FS). FS is a property that ensures that the compromise of long-term private keys does not reveal previous session keys. In other words, even if a long-term key is compromised, past session keys remain secure. FS is achieved by using ephemeral key pairs for each session and securely deleting the private values after the session ends. However, ephemeral Diffie–Hellman alone does not provide authentication. Therefore, the protocol must also incorporate an authentication mechanism, such as digital signatures or pre-shared keys, to prevent man-in-the-middle attacks. Liu et al. employed a Bi_2_O_2_Se-based TRNG to generate the ephemeral private values (random integers a and b) required for the Diffie–Hellman key exchange protocol. The inherent randomness and independence of the TRNG’s output ensure that these private values are unpredictable, which is a necessary prerequisite for achieving FS. In addition, the overall security of FS depends on the complete implementation of the protocol, including key management and secure erasure. This shift in design from ‘physical random sources’ to ‘intelligent security coprocessors’ signifies that memristor-based TRNGs are evolving towards a balance of low power consumption, high integration and adaptive information processing, offering a highly promising end-to-end solution for hardware security in the era of the IoT and edge computing.

It should be noted that a TRNG is not a directly deployable cryptographic component without rigorous statistical evaluation. In practice, the evaluation of a TRNG typically operates at two distinct levels: entropy-source validation and output-bit statistical testing. Entropy-source validation, as recommended in NIST SP 800-90B, assesses the physical source’s min-entropy, independence, and stationarity, whereas output statistical testing (e.g., NIST SP 800-22) checks the digitized bitstream for statistical defects. These two levels serve complementary but different purposes and should not be conflated. Among these metrics, four are of particular importance. Bias refers to the deviation of the output bit probability from the ideal value of 0.5. A biased bitstream reduces the effective entropy and thus compromises cryptographic strength. In practice, bias is typically quantified using the frequency test from the NIST SP 800-22 suite and can be mitigated through post-processing algorithms or bias-insensitive circuit designs. Autocorrelation measures the temporal dependence between bits separated by a given lag. For a truly random sequence, the autocorrelation coefficients at all nonzero lags should be statistically indistinguishable from zero. Autocorrelation is usually assessed via the autocorrelation function test, where the fraction of lags exceeding the confidence bounds should remain below a specified threshold, and it can also be analyzed through the runs test and serial test in the NIST suite. Independence requires that each bit in the output sequence be statistically independent of all other bits. This property is evaluated through multiple NIST subtests, including the cumulative sums test, the template matching test, and the approximate entropy test, which examine various types of dependencies within the bitstream from different perspectives. Stationarity means that the statistical properties of the TRNG output remain constant over time. A nonstationary entropy source may appear approximately random over short time windows but exhibit systematic drift on longer timescales. Stationarity is commonly assessed by testing the TRNG output under varying operating conditions and verifying that the statistical test results remain consistent.

It is important to emphasize that passing the NIST SP 800-22 statistical test suite by itself does not demonstrate true randomness, independence, or high entropy, as it merely indicates that, at the selected significance level, the bitstream does not exhibit obvious statistical patterns that would cause the selected tests to fail. Consequently, a comprehensive TRNG evaluation should report at least the following parameters: bias, autocorrelation, stationarity, min-entropy, sample length, significance level, number of test sequences, post-processing, and restart conditions. However, we found that the vast majority of original studies did not report these critical parameters. In light of these observations, we strongly advocate for the adoption of standardized and comprehensive reporting practices in future studies on memristor-based TRNGs. Specifically, we recommend that authors report at least the statistical properties of the raw bitstream without any post-processing, the complete test conditions including temperature, supply voltage, read voltage, and device aging state, the number of test sequences with the sample length per sequence and the significance level used in the statistical tests, a clear distinction between entropy source validation and output bit statistical testing, and the results of restart experiments to verify that the output is not simply reproduced after power cycling. We believe that the adoption of such standardized practices will greatly enhance the reproducibility, comparability, and credibility of memristor TRNG research, and will accelerate the translation of these promising prototypes from academic validation to practical hardware security solutions.

**Table 2:** Performance of true random number generators.

Entropy Source	Materials	Throughput Rate	NIST SP 800-22	Energy Consumption	Entropy Devices	Parallel Channels	Sampling Frequency	Post- Processing	Ref.
delay time/ delay time and relaxation time	Pt/Ag/Ag: SiO_2_/Pt	6 kb/s	15	~	4	1	≈11MHz	No	[68]
	Pt/HfO_2_/TiN	6 kb/s	8	~	5	1	~	No	[69]
	Pt/HfO_2_/TiN	16 kb/s	15	~	7	1	~	NFSR	[70]
	Cu_0.1_Te_0.9_/HfO_2_/Pt	32 kb/s	15	~	7	1	~	NFSR	[71]
	Ag/SiN_x_/n-Si	112 kb/s	15	~	18	2	~	Dual NESR	[72]
	Ag/a-Si/Pt	48 kb/s	14	~	5	1	~	No	[73]
threshold voltage	TiN/VO_2_/HfO_2_/TiN	5 Mb/s	~	1.8 nJ/bit	~	1	~	No	[78]
noise	Pt/Ti/NbO_x_/Pt	40 kb/s	15	5.23 nJ/bit	3	1	Self-clocked	No	[83]
	Ti/NbO_x_/Pt + Heater	800 kb/s	15	30 nJ/bit	2 × 8	8	Self-clocked	No	[84]
	TiN/OGL/MO/HfO_x_/TiN	41.7 Mb/s	15	~	>16,000	~	~	No	[86]
	Pt/Ti/TiO_x_/Al_2_O_3_/Pt/Ti	10 bit/s	15	~	4	1	~	No	[87]

### 2.2. Physical Unclonable Function Based on Memristors

Physical Unclonable Function (PUF) is a security technology that utilizes the random physical characteristics arising from the hardware manufacturing process to generate a unique, unclonable response to a given challenge. Much like a human fingerprint, it can be effectively applied to lightweight device authentication and anti-counterfeiting, as well as root key generation and storage. The microscopic origin of its physical randomness lies in the formation and breakage of conductive filaments within the resistive switching layer, which is a typical atomic-level random process. Upon application of an external electric field, Joule heating and concentration gradients drive the migration and aggregation of oxygen vacancies during the forming process and subsequent SET/RESET operations. Meanwhile, manufacturing uncertainties such as the local distribution of defect energy levels within the film, grain boundary orientation, and electrode interface roughness result in unpredictable and irreproducible variations in the position, number, cross-sectional morphology, and vacancy concentration distribution of the conductive filaments within each device. Distinct from TRNGs, the inherent random dispersion is static and spatial in nature. The entropy source must derive from device-to-device (D2D) variations introduced during the manufacturing process [67]. Once manufacturing is complete, these variations must remain stable and reproducible under variations in temperature, voltage stress, and time, to ensure that the same challenge always produces the same response. Uniqueness and reliability are the primary metrics. In this context, device variation serves as a ‘fingerprint’ to be preserved, rather than a dynamic signal to be sampled.

Since modern PUF chips possess abundant and diverse entropy sources, classification based solely on the type of entropy source becomes difficult. In general, PUFs can therefore be classified into weak PUFs and strong PUFs according to the number of challenge-response pairs (CRPs). A weak PUF has only a very limited number of CRPs and is typically used to derive a fixed cryptographic key. A strong PUF supports an exponentially large number of CRPs, making it infeasible for an attacker to exhaustively enumerate all responses within a finite time, and it is primarily employed for storage-free lightweight authentication. Building on this concept, a controlled PUF applies cryptographic algorithms to the raw responses of a strong PUF, thereby protecting the internal CRPs from direct exposure. It inserts a secure logic layer between the raw PUF and the external interface, which restricts access to the raw responses. However, this weak/strong dichotomy is somewhat coarse and does not fully reflect the implementation diversity of PUF designs. A more detailed classification can therefore be based on the bit generation method. Accordingly, PUFs can be divided into four categories according to the bit generation method: cell-comparison type, threshold-comparison type, column-comparison type, and delay-comparison type.

#### 2.2.1. Cell Comparison PUF

**Figure 8:**
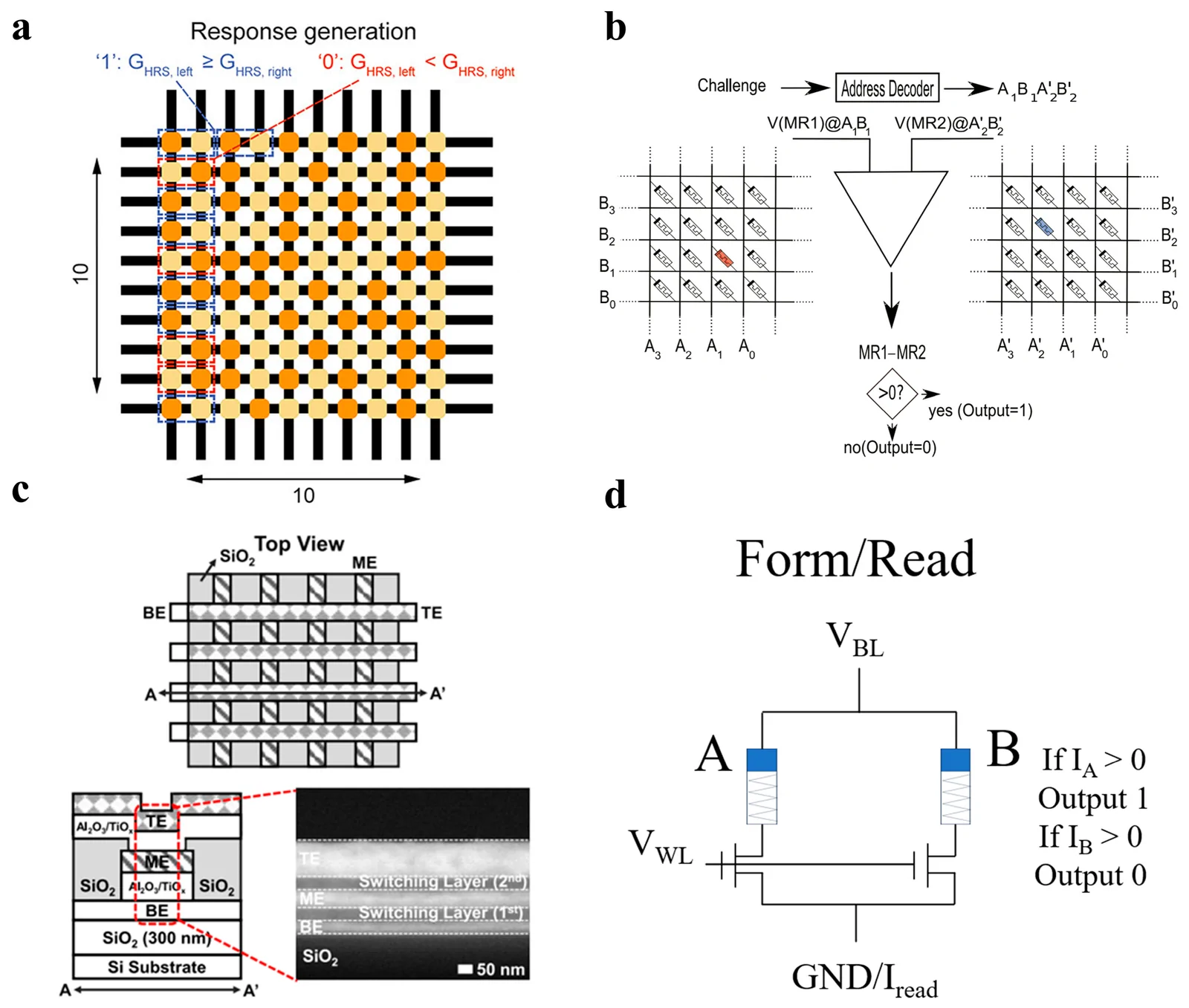
Cell comparison of Physical Unclonable Function (PUF). (**a**) Schematic diagram of reconfigurable key generation and extraction by comparing the HRS conductance between two adjacent memristor cells (differential pair) [93]. (**b**) Flowchart of differential comparison in a memristor crossbar array [94]. (**c**) Schematic diagram of Pt/Al_2_O_3_/Ti_x_O_y_/Ti_y_O_y_/Al/Pt memristor crossbar array and SEM images of each layer. The cross-sectional SEM image was obtained by cutting along the a–a’ section [95]. (**d**) Flowchart of the competitive comparison process.

Cell comparison PUFs generate bits by utilizing the differences in electrical parameters between different memristor cells within a memristor array to produce random responses. There are three methods for implementing comparisons between cells. The first method involves directly comparing the electrical parameters of two adjacent memristors, such as resistance [89] or subthreshold slope [90], to generate a response. As shown in Figure 8a, we illustrate the specific details using conductance values as an example. Following the challenge input, the HRS conductance values of the memristors are randomly distributed due to subtle random variations in each memristor. At this point, responses can be generated by directly comparing the conductance of adjacent cells. If the conductance of the left cell (GHRS, left) is greater than or equal to that of the right cell (GHRS, right), the differential pair is read as ‘1’. Otherwise, if the left conductance is less than the right conductance, it is read as ‘0’. However, in this approach, a pair of memristor cells can typically generate only one bit, resulting in a limited number of CRPs. Thus, Aitchison et al. proposed a new approach. By measuring the R-V curve and comparing resistances at different voltages, a single pair of memristors can generate a greater number of CRPs [91]. Nevertheless, with the advent of the AI era, attackers can collect CRPs to train AI models and subsequently launch attacks. Therefore, reconfigurable PUFs that can alter the physical state of the PUF via external inputs, thus refreshing or resetting its CRP mapping, are becoming increasingly important. Generally, such a reconfigurable function is achieved through electrical pulses. For example, the PUF designed by Yu et al. incorporates an additional dummy row. Upon reset and after comparing the current from the LRS with that of each cell in the same column, they overwrite the entire LRS, rendering it reconfigurable [92]. Moreover, Kim et al. employed a different approach using a 10 × 10 memristor array fabricated from two-dimensional chiral organic-inorganic hybrid perovskites. Drawing upon the differing photo-response of chiral perovskites to circularly polarized light (CPL), they achieved reconfigurability, demonstrating that photoelectronic memristors possess unique characteristics for PUF design [93].

The second method is illustrated in Figure 8b. When voltage is applied to two memristors connected in series, one of them turns on first due to differences in threshold voltage. The current at the intermediate node will generate a positive (or negative) pulse, and the polarity of the read current at this point can then represent a bit [94]. For example, a two-dimensional HfS_2_ memristor PUF generates a response by comparing the current difference between adjacent devices under opposite polarity read voltages [95]. Subsequently, Park et al. directly implemented a 3D stack of two series-connected memristors to form an array, as shown in Figure 8c. By utilizing the random distribution of HRS resistances to generate a response, they reduced the influence of leakage current on the bias distribution, significantly improving integration density and area efficiency [96]. The final method is the competitive comparison shown in Figure 8d. When voltage is applied to two selected memristors, one of them will be the first to successfully enter the LRS. The system then determines whether A or B has conducted to output a response of 0 or 1. If neither has conducted, the process is repeated. Examples include the multi-mode PUF [97] designed by Cui et al. and the memristor array designed by Li et al., which was also mentioned in the TRNG section [85,86]. Thus, cell comparison PUFs require no complex peripheral circuitry, resulting in lower area and power consumption. Furthermore, some designs support physical reconfiguration and concealment capabilities, allowing keys to be reset or the PUF’s identity to be concealed to defend against physical probing. Relative to the other PUF categories discussed below, cell-comparison designs offer the lowest circuit complexity but generate the fewest CRPs per unit area.

#### 2.2.2. Column Comparison PUF

**Figure 9:**
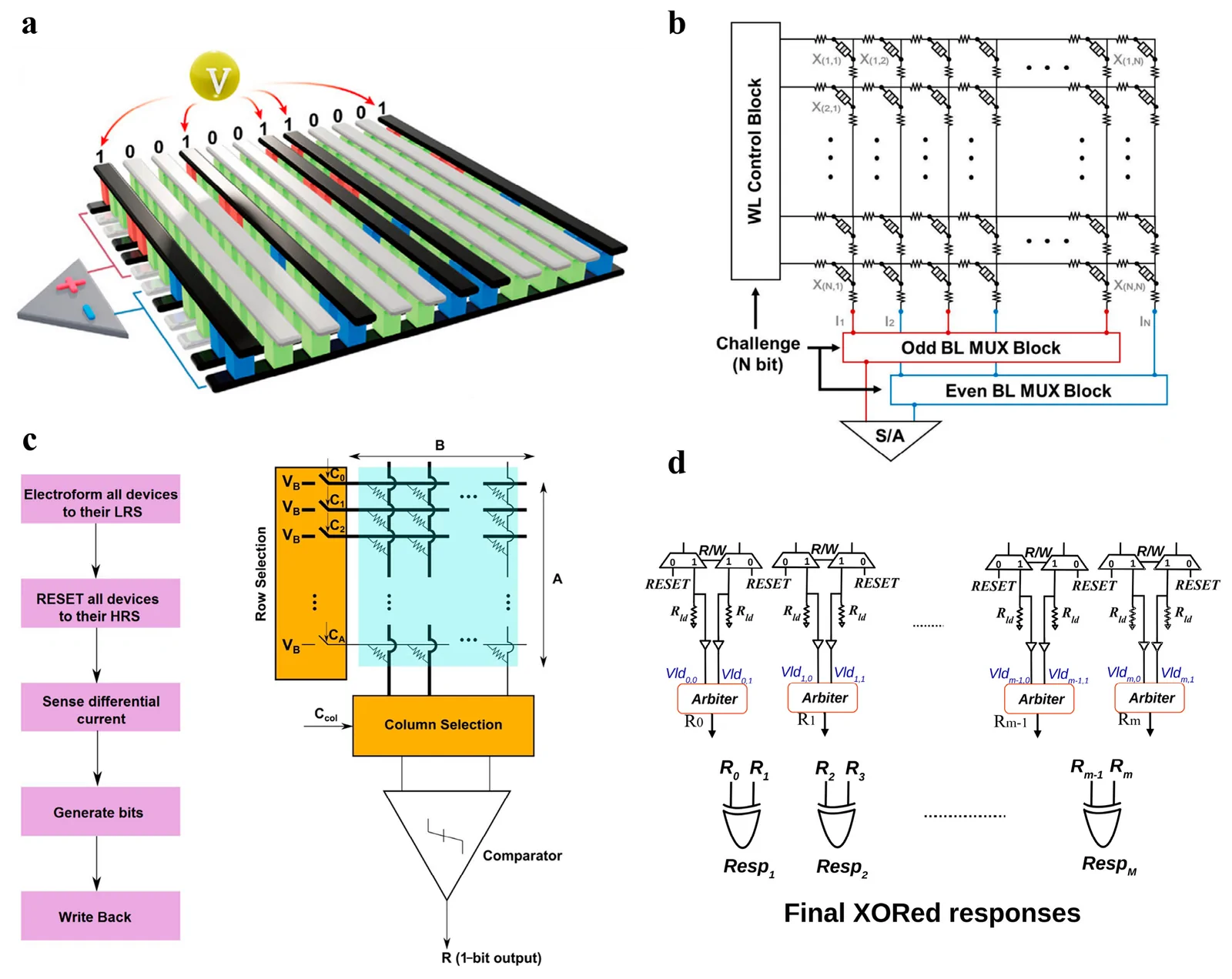
Column comparison of the Physical Unclonable Function (PUF). (**a**) Schematic diagram of a 12 × 12 Pt/AlO_x_/TiO_3_/Al_2_O_3_/Pt memristor array generating random bits, illustrating the logic of row selection and current comparison [98]. (**b**) Architecture of parity group comparison in an N × N cross-connected Pt/Ti/TiO_x_/Al_2_O_3_/Pt/Ti memristor array [100]. (**c**) Flowchart of the PUF bit generation process based on PrPyr[PbI_3_] memristors (**left**). Circuit architecture diagram of a 32 × 32 cross-array with differential sensing via low-offset comparators (**right**) [89]. (**d**) Schematic diagram of the read/write circuitry for an N × M XOR cross-memristor PUF that directly compares adjacent voltages [102].

Column comparison PUF chips utilizing memristor arrays have attracted increasing research interest in hardware security in recent years, due to their compact structure, high integration density and compatibility with CMOS processes. The core mechanism of such PUFs lies in applying a read voltage to the word lines in the array based on the challenge bits, where a challenge bit of ‘1’ for the kth row selects that word line by applying a read voltage, whereas a challenge bit of ‘0’ causes the current in that row to be ignored. Subsequently, the currents through the memristors on all selected word lines are summed in accordance with Kirchhoff’s current law. Owing to characteristics such as device-to-device randomness, probabilistic switching, and randomness in configured resistance values, the currents in different columns will ultimately differ. Therefore, comparing the currents in different columns allows bits to be generated. Different researchers employ different methods for column comparison, among which a common approach is to divide the array into left and right groups. As illustrated in Figure 9a, Na et al. designed a 12 × 12 PUF based on Pt/AlO_x_/TiO_x_/Al_2_O_3_/Pt oxide memristors. In this design, multiple word lines among the 12 can be arbitrarily selected and read simultaneously, although not all may be selected, yielding a total of 2^12^ − 1 = 4095 possible row combinations. Partition the 12-bit lines evenly into a left group of 6 columns and a right group of 6 columns. Then, 3 columns are randomly selected from each group to form column pairs. Since there are 20 possible column selection combinations on each side, 400 possible column pairing configurations exist, and a single chip can theoretically generate approximately 1.64 million CRPs [98]. Similarly, as technology advances, enhancing the security of CRPs through increased quantity alone is no longer sufficient; such technologies must also possess reconfigurability. For example, the PV3D3 polymer memristor PUF designed by Oh et al. exhibits excellent water resistance and flexibility [99]. In its strong mode, the entire array is divided equally down the middle into a left group and a right group, and the response bit is generated by comparing the cumulative total current in the left group with that in the right group. Having fabricated a 32 × 32 array, they were able to generate 2^32^ CRPs. Furthermore, through the application of excessive electrical stress to the memristors, the internal conductive filament paths can be actively disrupted and reshaped, thereby achieving reconfigurability. The ability to generate a large number of CRPs while maintaining reconfigurability is a fundamental prerequisite for the future large-scale practical application of PUFs.

In addition to the left–right grouping strategy, certain designs partition the array into odd and even groups, such as the TiO_x_/Al_2_O_3_-based memristor PUF shown in Figure 9b [100]. Depending on the challenge, one column is selected from the odd group and one from the even group, and the signals are then compared via a sensitive amplifier. Other studies, however, avoid such grouping altogether, as in the one-dimensional halide perovskite memristor PUF mentioned in the previous section. Figure 9c illustrates its strong mode. Initially, all memristor cells are in the LRS, after which the RESET operation returns all memristors to the HRS, and the bit is output by directly comparing the HRS currents of two selected columns based on the challenge, a strategy that significantly increases the number of CRPs [89]. Earlier research relied on directly comparing adjacent columns to output the bit. Uddin et al. conducted extensive simulation and experimental studies in this area. They utilized the randomness of SET times to apply fixed-width pulses to the selected row so that only some memristors switch to the LRS, and compared the currents of two adjacent columns [101], or added load resistors at the end of each column to compare the load voltages of adjacent columns, with the corresponding peripheral circuit shown in Figure 9d [102,103]. Similarly, by comparing adjacent load voltages, Khan et al. proposed a dual-memristor cross-array PUF. The alternating output of memristor arrays with highly mismatched electrical characteristics significantly enhanced the ability to resist machine learning modeling attacks [104]. Although column comparison PUFs differ in their method of selecting columns, the underlying bit generation principle remains largely the same. While issues such as systematic spatial current bias and leakage current severely affecting read accuracy may arise as the array scale increases, column comparison PUFs are capable of generating a significantly larger number of CRPs, making them highly suitable for robust PUFs. These characteristics render them particularly well-suited for applications where security, lightweight design, and scalability are of the utmost importance. Compared with cell-comparison designs, column-comparison PUFs offer orders of magnitude more CRPs but require more complex peripheral circuitry to handle current summation and comparison.

#### 2.2.3. Delay Comparison PUF

Delay comparison PUFs typically require the integration of memristors with MOS transistors, yet the hybrid integration process for memristors and CMOS is not yet mature, presenting issues such as high fabrication costs and long lead times. Consequently, research on this type of PUF remains at the simulation verification stage, with the most prominent contributions coming from the teams led by Mathew and Loong. A series of works by the two teams is based on TiO_2−X_/TiO_2_ memristors. The PUF was first designed by Mathew’s team in 2015, which generates a 1-bit response by comparing the signal arrival times of two parallel paths, where a multi-stage delay path is formed by connecting the memristor and MOSFET in series. The challenge bit controls the on/off state of the switch to alter the total impedance of each path, and a D-flip-flop arbitrates which signal arrives first to output the bit [105]. Then, an improvement based on this circuit was proposed the following year, in which the MOSFETs and memristors were connected in parallel rather than in series to resist cryptanalytic attacks based on challenge partitioning, while retaining the ability to resist machine learning modeling attacks [106]. In the same year, the team led by Loong proposed another improvement by inserting multiple arbiters into the paths to achieve parallel multi-bit response output, significantly increasing the challenge-response space, and by replacing D flip-flops with SR latches to improve path symmetry and reduce response bias [107]. Furthermore, in 2015, Mathew’s team designed a memristor–CMOS hybrid XOR gate based on this memristor, cascading them to form multi-stage delay paths where the challenge determines the transmission direction of the signal edge. The combination of signal edge direction and path delay results in a non-linear relationship between the response and the challenge, making it difficult to fit with a linear model [108]. Other architectures include the ring oscillator PUF, a special type of delay-comparison PUF, which generates a response by comparing the frequencies of different oscillators (i.e., the reciprocal of the loop delay). The memristor can replace the load resistor in circuits, exploiting the non-linear resistance and variation characteristics of the memristor to enhance the randomness of oscillation frequency and thereby improve the traditional RO-PUF [109,110]. The reconfigurable TRGP architecture [111] controls the PMOS transistor inverter delay in PUF mode and adjusts the phase difference output response of the two oscillators. Most of the above achievements remain at the experimental simulation stage, with a severe lack of concrete hardware implementations, and future research could attempt to bridge this gap. Distinct from the spatial comparison schemes described above, delay-comparison PUFs exploit temporal randomness, offering the advantage of compatibility with standard CMOS timing analysis tools, but their practical deployment is currently hindered by the lack of mature memristor–CMOS integration processes.

#### 2.2.4. Threshold Comparison PUF

**Figure 10:**
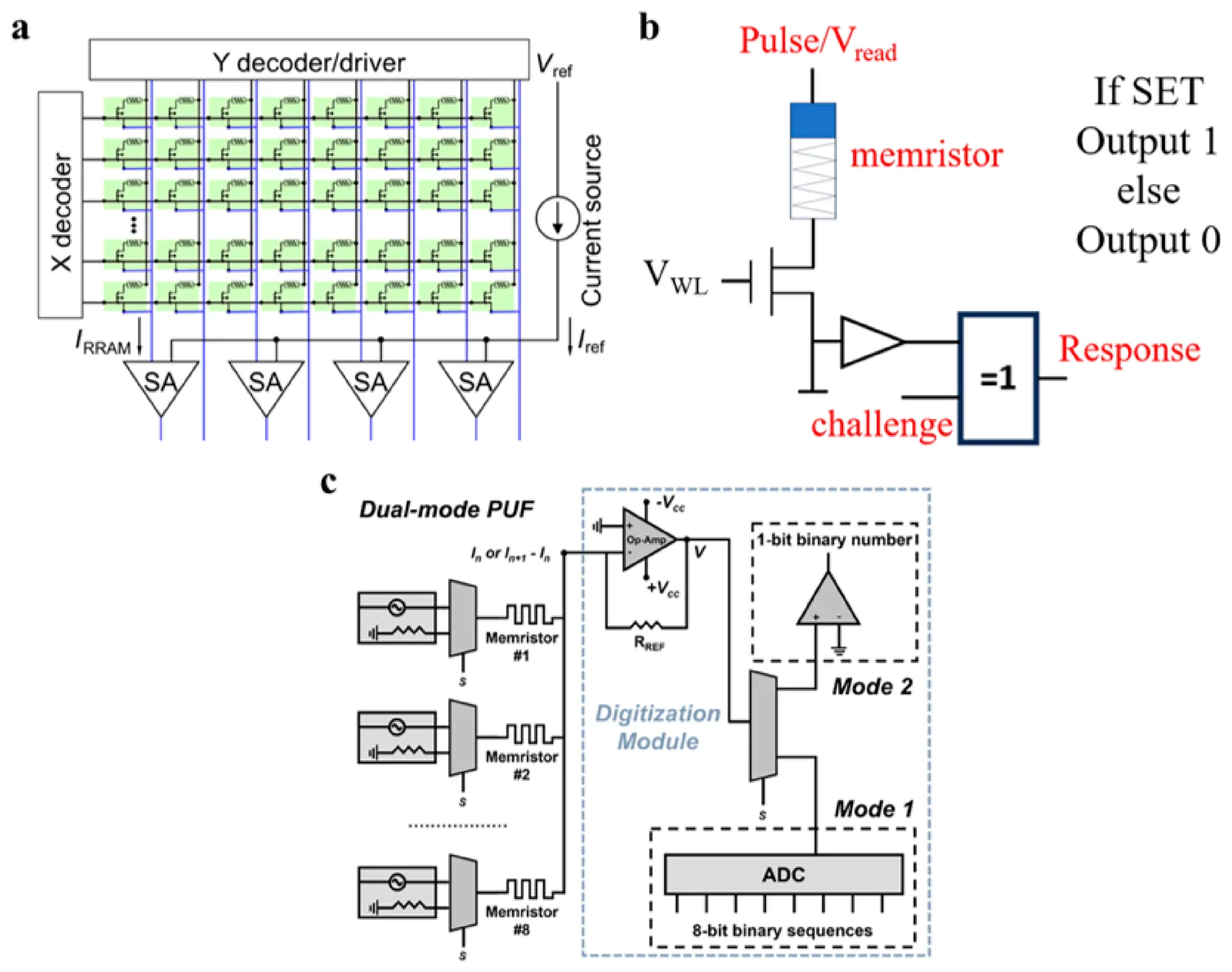
Threshold comparison of a Physical Unclonable Function (PUF). (**a**) Schematic diagram of the TiN/TaO_x_/HfO_x_/TiN memristor PUF structure [114]. (**b**) PUF cell that generates a response by utilizing process variations in memristor write times. (**c**) HfS_2_-based memristor PUF with dual modes [95].

In terms of the specific implementation of bit generation, threshold comparison PUFs have undergone considerable evolution, ranging from single-threshold to multi-threshold, from single-ended to differential, from electrical to optical, and from the time domain to the current/voltage domain. The most fundamental approach is single-threshold current comparison. As shown in Figure 10a, the currents of all cells are read, and the median value is set as the reference, so that the current from each cell is compared with the median to generate a response. For example, the ITO/IGZO/TaN memristor PUF designed by Park et al. employed additional dual-wavelength optical stimulation to modulate the conduction mechanism, enhancing randomness and discriminability [112]. However, complex on-chip reference current search circuits increase both power consumption and area. Employing a W/Ag/MgO/Ag/W memristor, Sun et al. discovered that switching probability can be controlled by adjusting the Ag sputtering power, with approximately 50% of the devices in the array switchable when the Ag sputtering power is 70 W. Identical scanning voltages are applied to the entire array together with a fixed reference current. If a device is switched on (current exceeds the reference value), it outputs a ‘1’ bit. Conversely, output “0” [113]. Notably, the TiN/TaO_x_/HfO_x_/TiN memristor PUF designed by Gao et al. in 2022 innovatively incorporated a concealment function [114]. Programming the memristor as LRS by applying a single SET pulse masks the original conductive filament morphology. At this point, the PUF bits read are close to all ‘1’, preventing attackers from obtaining a valid key. Through the application of a single RESET pulse, the ‘cycle-to-cycle correlation’ characteristic of the memristor is exploited to erase newly generated dynamic oxygen vacancies and restore the conductive filaments to their initial state. The emergence of this concealment capability has significantly enhanced the security of PUF designs and is driving memristor-based PUF chips towards practical application.

By contrast, time threshold comparison PUFs fix the write pulse width and utilize the random distribution of the actual minimum SET time of the device to determine whether resistance switching occurs, thereby generating the response. As shown in Figure 10b, a memristor that fails to SET during the pulse duration remains in the HRS and produces an output of 0, whereas successful SET within this duration drives the device into the LRS and yields an output of 1. This time-threshold comparison scheme was first proposed, simulated and experimentally verified by Rose et al. [115,116]. Subsequently, Mazady et al. were the first to experimentally verify this time-threshold comparison scheme using ZnO nanowire memristors [117]. Multi-level threshold comparison schemes divide the analogue voltage into multiple intervals, enabling a single device to contribute multiple-bit responses and significantly enhancing CRP density [118]. As shown in Figure 10c, the HfS_2_-based dual-mode PUF mentioned earlier provides an example of this approach, since in Mode 1 the LRS current of each memristor is converted to a voltage and quantized into a multi-bit binary number by an analogue-to-digital converter (ADC), fully leveraging the current differences between devices [95]. Threshold comparison PUFs are relatively sensitive to environmental factors such as temperature, as they rely on the internal thresholds of memristors. They also place stringent demands on process control and circuit design and are therefore classified as a technically demanding category of PUF. However, directly utilizing the inherent memristor thresholds for comparison eliminates the need for space-consuming and power-hungry CMOS comparators, considerably reducing design complexity and chip area, while multifunctional integration enables the efficient reuse of hardware resources. Among the four PUF categories, threshold-comparison designs offer the most compact structure per bit and the highest CRP density, but their environmental sensitivity poses the greatest challenge for practical deployment under varying operating conditions.

To systematically compare the performance of different PUF schemes, Table 3 summarizes the reported values of the reviewed PUF types on key metrics such as uniqueness, reliability, and area overhead. Because the individual studies employ different test conditions, technology nodes, and definition methodologies, the values in the table should not be interpreted as an absolute ranking. The principal objective of this table is to illustrate the typical magnitude ranges that each PUF category achieves for these metrics, thereby enabling readers to form a holistic view of the performance distribution across the various technology approaches.

#### 2.2.5. Applications of PUF

**Figure 11:**
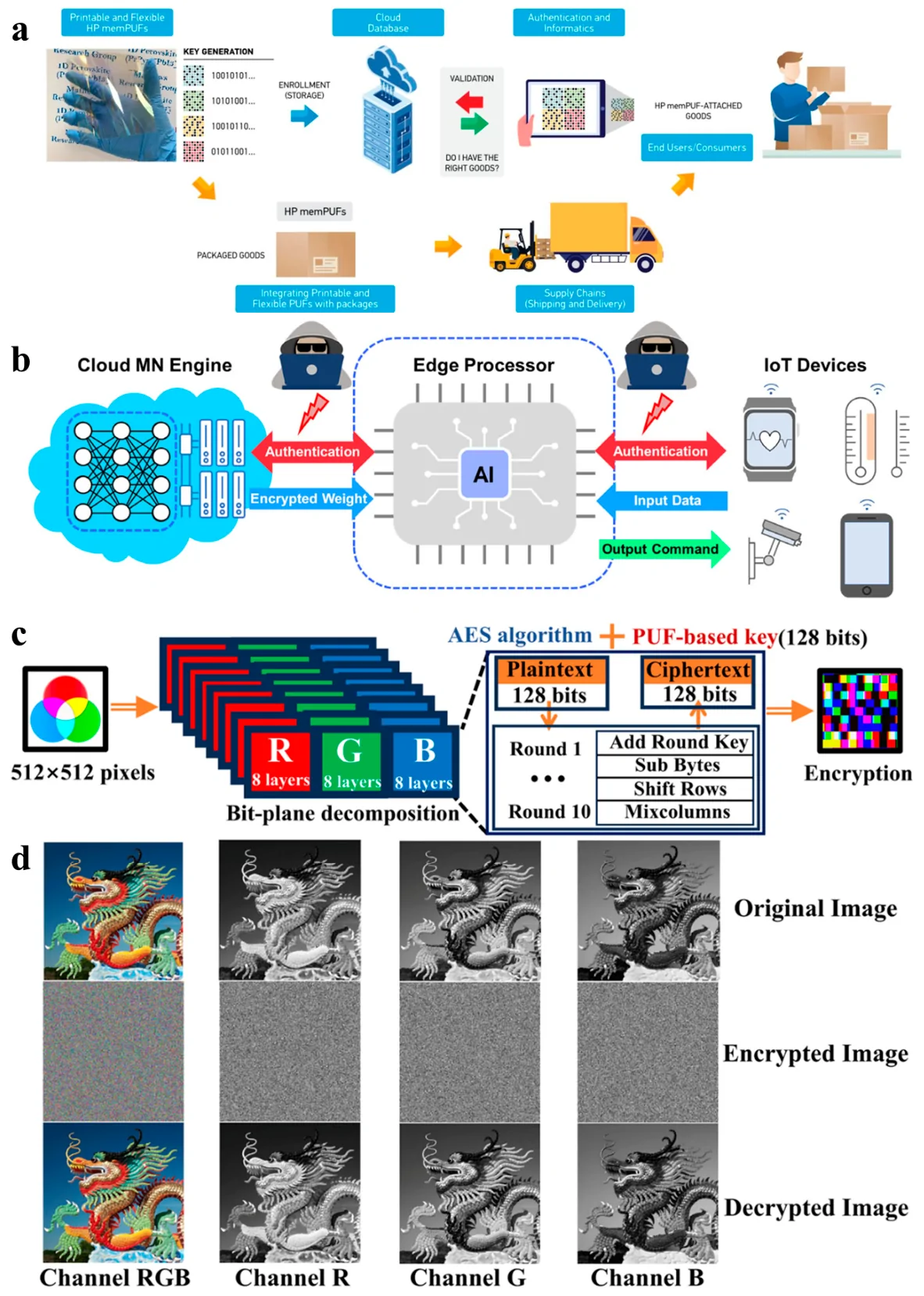
Applications of Physical Unclonable Function (PUF). (**a**) Concept schematic of product authentication [89]. (**b**) The security system of the IoT network consisting of the cloud ML server, edge processor with a neuromorphic processor, and things with various sensors [99]. (**c**) Schematic illustration of combined hardware and software for image encryption [119]. (**d**) The original, encrypted, and decrypted images of Chinese Loong and the corresponding three-channel components [119].

Memristors, owing to the inherent randomness of their resistive changes, have become an ideal medium for constructing PUFs, bridging the gap between physical entropy sources and digital keys for hardware security. The core advantage stems from the fact that different materials and switching mechanisms can generate multi-dimensional randomness. As such, these rich sources of entropy can enable various security scenarios, including device authentication, system-level security protocols, and data encryption. Most PUF applications are at the device-authentication level, and although authentication methods vary, the underlying principle is to use process variations to provide a hardware fingerprint. An example is the halide perovskite memristor array designed by John et al. The authentication process is illustrated in Figure 11a. Manufacturers attach PUF chips to product packaging and register each PUF ID (initial key) with a secure cloud database. End users (consumers or supply chain nodes) scan the product to initiate an authentication request to the cloud. Cloud services then issue a random challenge. The product PUF generates a response, which the cloud compares against the expected response in the database to verify the authenticity of the product. Additionally, the PUF array can store supplementary information such as product expiry dates and logistics routes [89].

Concerning system security protocols, the memristor-based PUF designed by Oh et al. [99] and integrated into a neuromorphic system proposes a mutual authentication protocol. As shown in Figure 11b, the cloud and the IoT device first perform mutual authentication by exchanging CRPs via a robust PUF. Once authentication is successful, the cloud uses this CRP as a symmetric key to encrypt the neural network weight matrix for transmission. After decryption, the device writes the data into the memristor synapse array for inference. Concurrently, the same protocol is employed for mutual authentication between the edge processor and the sensor, which effectively prevents man-in-the-middle attacks that might tamper with weights or inject false data, ensuring the integrity and confidentiality of the entire data link from the cloud to the edge. Meanwhile, the Ta/WTiO_x_/Pt memristor array PUF proposed by our team enables image encryption and decryption coordinated between software and hardware [119]. Figure 11c illustrates the encryption and decryption process involving the AES algorithm and the memristor array. A 512 × 512-pixel image, comprising three channels (red, green, blue), is decomposed into 24-bit planes. AES encryption is performed on each 128-bit plaintext block. Once all blocks have been encrypted, the encrypted bit planes are recombined to form the encrypted image. The AES algorithm is integrated into a separate module, providing software support for encryption and decryption. As shown in Figure 11d, an original RGB image and three component images of a Chinese dragon are presented. After the memristor array first generates a 128-bit PUF key and the AES algorithm performs the collaborative encryption operation, recognition of the encrypted image becomes completely impossible. Following the decryption process, the decrypted image is identical to the original. These developments demonstrate that memristor-based PUFs are evolving from mere identity generation tools into comprehensive hardware security solutions that integrate authentication, protocol protection, and cryptographic operations.

### 2.3. Other Hardware Solutions

In addition to TRNGs and PUFs, memristor-based hardware security research has also produced a range of other solutions. These can be classified into three categories according to their developmental maturity and design philosophy: architectural design and simulation-based schemes, physical implementation and digital logic encryption, and emerging security paradigms with system-level integration. The classification reflects a progressive trajectory from theoretical exploration through physical realization to paradigm innovation, mirroring the broader evolution of the field.

#### 2.3.1. Architectural Design and Simulation-Based Schemes

The first category focuses on architectural design and simulation-based verification, emphasizing the exploration of security defense mechanisms using memristor models, without involving the fabrication of actual devices. This category of research can be further subdivided into two sub-directions: the first concerns the hardware acceleration of traditional cryptographic algorithms and key generation. For example, Rady et al. [120] utilized a memristor TEAM model to design a time-domain analogue-to-digital converter read circuit, extracting the device’s intrinsic process variations to generate an AES-128 symmetric key. The second category comprises system-level protection strategies against physical attacks. For instance, Gong et al. [121] utilized the retention loss characteristics during memristor read operations to embed an analogue-domain cross-array at the output of the scan chain, obfuscating the scan response through matrix multiplication, whilst employing linear least-mean-squares estimation and a periodic refresh mechanism to ensure data recovery for legitimate users. Yang et al. [122] focused on the protection of intellectual property in neuromorphic computing systems. They utilized the aging effect of memristors to cause inference accuracy to degrade non-linearly with the number of operations, preventing attackers from reverse-engineering proprietary models by collecting input-output pairs. Singh et al. [123], on the other hand, addressed data fingerprinting attacks on SRAM by integrating memristor-assisted paths into 6T SRAM. Through in-memory XOR logic, they achieved single-cycle data flipping across the entire array, thereby eliminating the NBTI electrical fingerprints generated by long-term static data patterns and, at the physical level, cutting off attackers’ ability to extract residual information. Although such research has not yet involved actual chip fabrication, its value lies in providing highly feasible architectural references for subsequent hardware implementation.

#### 2.3.2. Physical Implementation and Digital Logic Encryption

**Figure 12:**
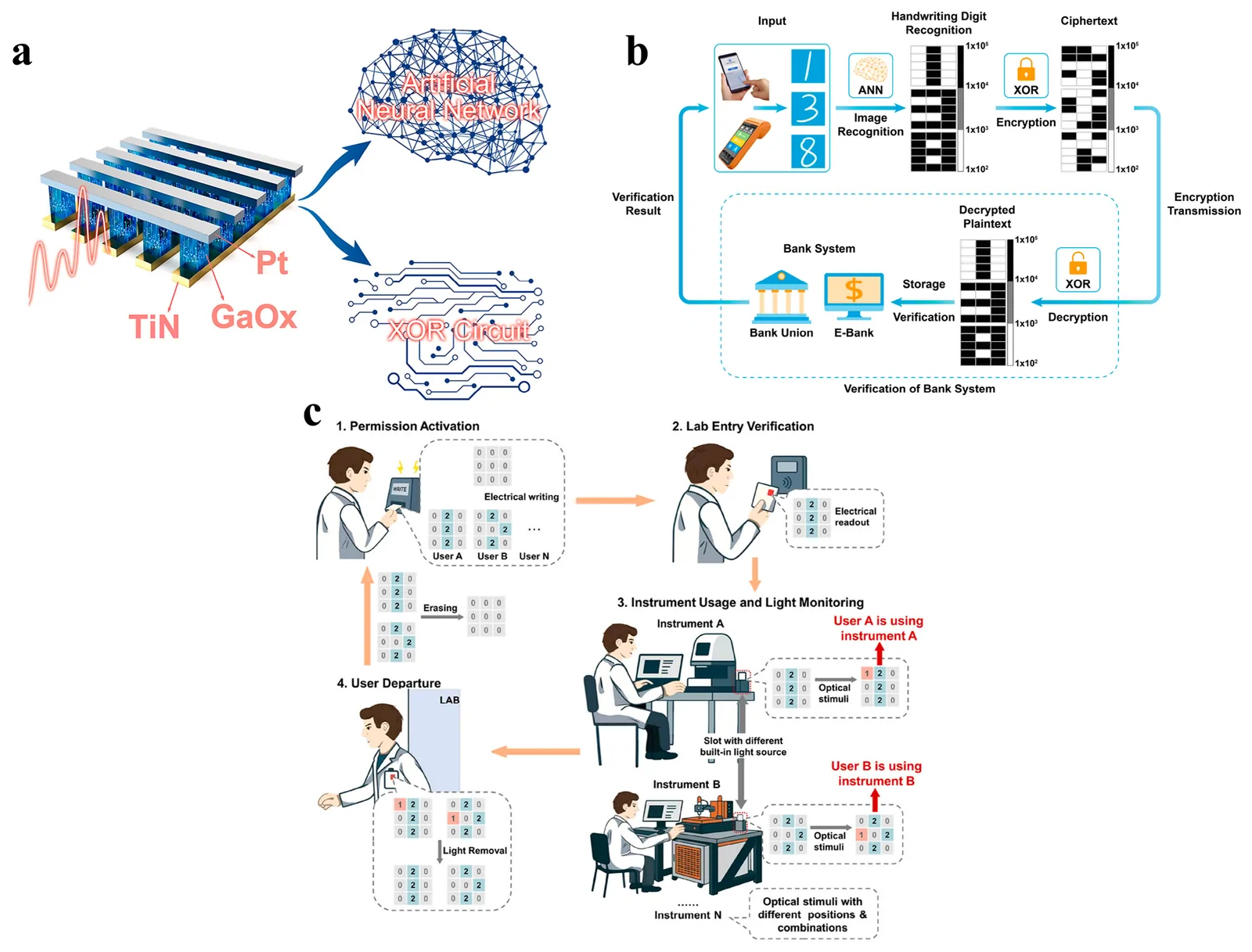
Other Hardware Solutions based on memristors. (**a**) Schematics of the Pt/GaO_x_/TiN device [124]. (**b**) The process diagram of the data security system based on ANN and XOR logic for banking and e-commerce applications [124]. (**c**) Practical operation flow chart based on Ag/PMMA/PEA_2_MAPb_2_I_7_/ITO optoelectronic memristor [129].

The second category focuses on the physical realization of nanodevices and digital logic encryption, advancing memristors from simulation models to actual chip fabrication, and utilizing their multistate storage capabilities or reconfigurable logic functions to implement traditional digital encryption algorithms. The category of research is based on mature material systems such as transition metal oxides and has verified the on-off ratio, endurance, and retention characteristics through electrical testing. In terms of functional implementation, as shown in Figure 12a, our team utilized a Pt/GaO_x_/TiN memristor array to construct an artificial neural network for handwritten digit recognition, combining this with single-device XOR logic to perform data encryption and decryption. The specific procedure is shown in Figure 12b. After the user enters their password by handwriting, an ANN built on a memristor array performs precise recognition. The memristor array then generates a truly random key to perform an XOR encryption operation on the ciphertext, transforming it into meaningless ciphertext. Once securely transmitted to the bank’s cloud system, the same key is used to decrypt the ciphertext, accurately restoring the original ciphertext. Finally, the result is verified against the database and returned to the mobile device. This system eliminates the risk of data leakage associated with traditional scrambled keypads and, at the hardware level, provides end-to-end data protection—from secure input and efficient encryption and decryption to transmission verification—effectively warding off external threats such as software cracking and channel eavesdropping [124]. Yan et al. [125] constructed full adders and AND/OR/XOR logic gate circuits based on Ag/ZrO_2_/BiFeO_3_/Ti devices, achieving 8-level hierarchical image encryption and Morse code covert communication. Sun et al. [126], meanwhile, utilized the eight stable resistance states of HfAlO_x_ memristors for multi-state random mapping encryption, achieving a recognition rate of 98.1% following decryption verified by a CNN. The above research indicates that memristor-based hardware security has progressed from the ‘architectural design’ stage to the ‘physical chip’ stage. However, its fundamental security still relies on the logic of digital circuits and algorithmic complexity.

#### 2.3.3. Emerging Security Paradigms and System-Level Integration

The third category focuses on emerging security paradigms and system-level functional integration, representing the cutting edge of memristor-based hardware security. Its core lies in overcoming the limitations of digital Boolean logic, deeply exploring the unique non-linear, random, and transient characteristics inherent in memristors and associated physical fields, and constructing an entirely new security paradigm that is difficult to achieve with traditional CMOS technology. In addition, Cheong et al. [127], utilizing a Pt/Ta_2_O_5_/Mo metal-cluster memristor, leveraged its random switching characteristics to generate encryption keys within a 1T1R array. By utilizing its analogue storage capability to perform vector-matrix multiplication on ciphertext, they achieved hardware acceleration of homomorphic encryption for the first time. Furthermore, while on-chip key storage may reduce the risk of direct key exposure, it does not by itself provide protection against side-channel attacks such as power, electromagnetic, timing, or fault-based attacks. Appropriate countermeasures would still be required to achieve robust side-channel resilience. Kuka et al. [128] constructed a Chua chaotic circuit using memristors, achieving physical-layer encryption for near-field wireless communication through chaotic waveform synchronization. The intrusion of a third-party device disrupts the chaotic state, thereby blocking communication. Du et al. [129] utilized perovskite optoelectronic devices to integrate self-powered photodetection and resistive-switch storage within a single cell. By employing ternary logic states, they achieved real-time, dual-factor (optical/electrical) security monitoring. A practical operation flowchart is shown in Figure 12c. First, by employing a +0.5V electrical pulse, specific pixels are switched from the initial high-impedance state (logic “0”, ≈10 ^12^ A) to a stable low-impedance state (logic “2”, ≈10 ^6^ A), forming a user-exclusive electrical access pattern as a static ID card. During access control verification, a 0.2V read voltage scans the array and only allows passage if the measured current state distribution exactly matches the stored template. After entering the laboratory, the user card is inserted into a specific instrument slot, and the built-in light source projects a unique optical pattern (600 nm illumination) onto the array. The illuminated pixels instantly transition to the photoinduced transient (logic “1”, ≈10 ^9^ A), while the original “2” state pixels remain unchanged, forming a composite spatial distribution of “1” and “2” coexisting on the 3D current map, enabling real-time, unique identification of who is currently using which device. After the user leaves and removes the card, the lighting stops, and the entire “1” phase is automatically erased to restore the “0” state. The system reverts to the base state that only contains the electric writing pattern, or the new code is rewritten by electrical operation to erase and rewrite the new code. The entire process utilizes ternary encoding and an ultra-low power consumption of only 3.8 fj/bit, enabling reconfigurable permission activation, authentication, device occupancy monitoring, and automatic reset on the same hardware without complex external logic, eliminating the physical risk of a single factor being forged or bypassed. Yalagala et al. [130], on the other hand, approached the problem from the perspective of data destruction. They utilized a water-soluble MgO-PVP-graphene composite to fabricate flexible transient memristors. By triggering the device via a mobile app using Bluetooth, it completely dissolved in deionized water within 4 s, fundamentally eliminating the risk of data theft through physical disassembly. Taken together, this type of research extends the boundaries of security protection from the logical domain to the physical and material domains, opening up an entirely new technological dimension for high-energy-efficiency, highly robust hardware security protection in IoT edge devices.

Collectively, the three categories of other hardware solutions, architectural simulation, physical implementation, and emerging paradigms represent successive stages of technological readiness and conceptual innovation. While the first category provides low-cost validation platforms, the second demonstrates tangible chip-level functionality, and the third explores fundamentally new security primitives beyond digital logic. Their coexistence reflects the multifaceted nature of memristor-based hardware security research, where immediate engineering challenges and long-term vision are pursued in parallel.

Although the reliability and maturity of memristors still need to be further improved, the aforementioned achievements—marking a shift from single-function to multi-functional integration, from passive defense to active countermeasures, and from discrete components to system-level integration—have amply demonstrated that memristor-based hardware security solutions can effectively overcome the energy consumption and security bottlenecks of traditional von Neumann architectures. They offer a new pathway for lightweight, low-power, and highly robust physical-layer protection in future high-security scenarios such as the Internet of Things, edge computing, and financial payments.

## 3. Conclusions

Based on the comprehensive survey presented above, the following conclusions can be drawn. Memristors, endowed with intrinsic stochasticity, nonvolatility, and in-memory computing capability, offer a physical foundation for hardware security that is qualitatively distinct from conventional CMOS solutions. Across the surveyed literature, two primary implementation strategies have emerged: TRNGs, which exploit temporal random jitter or threshold voltage statistics, and PUFs, which leverage device-to-device resistance or timing variations. These two approaches share the same underlying stochastic mechanism at the physical level and diverge only in the manner of information extraction. On this basis, extended schemes, including nonvolatile secure memory, logic obfuscation, and physical attack detection circuits, further broaden the functional scope of memristors from key generation to system-level security protection.

A systematic performance comparison yields several key observations. For TRNGs, delay-time-based designs offer high intrinsic randomness quality with minimal post-processing but suffer from limited throughput. Threshold-voltage-based designs achieve higher speed at the cost of environmental sensitivity. And noise-based designs deliver the best throughput yet demand the most sophisticated readout circuitry. For PUFs, cell-comparison and column-comparison types exploit spatial randomness across arrays, with the latter providing orders-of-magnitude more CRPs at the expense of increased peripheral complexity. Delay-comparison types utilize temporal randomness but are constrained by immature memristor–CMOS integration. Threshold-comparison types offer the most compact structure per bit but exhibit the greatest environmental sensitivity. Notably, no universal solution dominates across all metrics. The trade-offs among different paths are ultimately dictated by the priority ordering of throughput, power, area, and environmental robustness for specific applications. Furthermore, most existing studies remain at the level of single-device or single-array performance verification, and the absence of system-level evaluation frameworks hinders fair cross-comparison among different approaches.

The integration of memristor-based PUFs and TRNGs into large-scale crossbar arrays introduces several practical non-idealities that must be considered, as they can significantly degrade both the performance and the security of the primitive. Sneak-path currents through unselected cells distort read operations and reduce the reliability of response extraction [92,131,132]. Line resistance along word and bit lines, together with the resulting IR drop, produces spatial gradients across the array, leading to position-dependent variation in the readout [133]. For column-comparison PUFs in particular, this IR-drop-induced variation is systematic rather than purely random, generating a position-dependent bias that skews the response distribution away from the ideal 50% and directly degrades uniformity. More critically, this systematic spatial structure can be exploited by machine-learning-based modeling attacks, as it introduces predictable correlations that an adversary can learn from a limited set of CRPs [134]. Beyond IR drop, other factors further impact security metrics: selector requirements add device complexity and may limit array size [135]. Read disturb causes long-term drift of the entropy source, threatening reliability [136]. The overhead of ADCs and sense-amplifier offsets introduces additional bit errors and area/energy consumption that affect the overall resource efficiency [137,138,139]. These crossbar-specific issues must therefore be analyzed holistically, with a clear link between each physical non-ideality and the security property it compromises.

Beyond these performance considerations, the transition from research to mass production faces three structural technical obstacles. First, memristor security performance depends on the statistical behavior of nanoscale conductive filaments, which is sensitive to temperature, voltage stress, and device aging. Ensuring response stability across varying environmental conditions while maintaining sufficient randomness constitutes an inherent contradiction at the device physics level: higher randomness tends to be accompanied by greater response dispersion, and these two aspects cannot be simultaneously improved through a single process optimization. Second, although back-end integration of memristors with advanced CMOS processes is feasible at the technological level, thermal budget and material compatibility constraints have yet to receive systematic experimental validation. For example, some oxide memristors exhibit oxygen diffusion problems following high-temperature annealing. Consequently, the temperature of CMOS back-end processes is typically limited to below 400–430 °C. Meanwhile, some chalcogenide or single-crystal oxide devices require deposition or annealing temperatures exceeding 600 °C, which risks compromising the performance of the underlying transistors. This is particularly critical when security IP is embedded into large-scale system-on-chips, where reliability and yield data are still limited at present. Third, existing schemes generally lack co-design considerations with upper-layer cryptographic protocols. Quantitative mapping between the output quality of security primitives and the security requirements of application scenarios has not been established, resulting in a disconnect between hardware security modules and system-level security architectures.

To advance memristor-based security solutions toward practical engineering deployment, future research must achieve substantial breakthroughs in the aforementioned obstacles. At the device level, the primary bottleneck remains the precise control of the resistive switching behavior of memristors. Large AI models can be employed to explore how doping engineering and interface modulation govern the evolution of conductive filament formation kinetics, thereby enabling the on-demand identification of optimal materials and dopants for constructing TRNGs with high entropy quality or PUFs with high reliability. Furthermore, greater attention can be devoted to novel memristors that operate through multi-physics coupling, such as optoelectronic co-modulation, where the multidimensional control capability allows multiple functions within a single device, enhancing randomness in the TRNG mode while locking in stability in the PUF mode.

At the circuit level, the principal technical challenge for practical application is to simultaneously improve the efficiency and precision of entropy extraction and the resilience against attacks while minimizing area overhead. For large-scale arrays, exploring time-to-digital converters as a replacement for conventional ADCs can reduce both area and power consumption while boosting entropy extraction efficiency and preserving readout accuracy. To strengthen attack resistance, circuits capable of dynamically refreshing the challenge-response pair mapping can be developed, actively renewing the fingerprint once suspicious attack behavior is detected. In addition, to counteract the systematic bias caused by IR drop and spatial gradients, column-to-column differential readout architectures and on-chip adaptive calibration circuits can be introduced. By comparing currents from adjacent columns or symmetric positions rather than relying on a global reference, such approaches effectively cancel position-dependent systematic bias.

At the system level, hardware security primitives must be tailored to the specific application. An application-oriented security evaluation framework should be established to map metrics such as TRNG throughput and power consumption and PUF uniqueness and reliability onto a quantifiable system security strength, thereby enabling the design of appropriate redundancy without wasting performance in a given application context. Only by bridging the entire chain from device physics to system integration can memristors evolve from laboratory curiosities into fundamental building blocks of trusted hardware.

## Figures and Tables

**Table 1:** Comparison with the main previous reviews.

	Ref. [67]	Ref. [40]	This Work
Scope	TRNGs and PUFs	TRNGs, PUFs and chaotic circuit	TRNGs, PUFs and other memristor-based security schemes
Covered period	By 2018	By 2020	By 2025
Classification method of TRNGs	Entropy source	Diffusive memristor and oxygen vacancy transformation	Entropy source
Classification method of PUFs	PPUF, weak PUF, strong PUF and double-layer PUF	Application and architecture	Bit generation
Comprehensive Performance table	No	No	Yes
Discuss the application	Authentication and key generation	Image encryption and security protocols	Authentication, image/voice encryption and security protocols
limitations	The challenges at the device and circuit level	Crossbar non-idealities and power consumption	Environmental sensitivity and aging, crossbar non-idealities and system-level deficiency

**Table 3:** Performance of physical unclonable function.

Bit Generation	Materials	Uniqueness (Inter-HD)	Reliability (BER)	Methods	Ref.
Cell comparison	PrPyr[PbI_3_]	≈48.3%	≈2.33% (100 cycles)	Fabricated devices with external instruments	[89]
	Al_2_O_3_/TaO_x_	≈50.1%	≤1% (0–80 °C and ±10% supply voltage fluctuation)	Software processing of measured data	[92]
	2D chiral perovskite	≈49.8%	≈5.1% (after reconfiguration)	Fabricated devices with external instruments	[93]
	~	≈50.2%	≈5.6%	Simulation	[94]
	HfS_2_	≈49.7% (mode 1) ≈52.7% (mode 2)	≈5% (mode 1) ≈0% (mode 2 within 10^5^ s)	Fabricated devices with external instruments	[95]
	TiO_x_/Al_2_O_3_ (3D stacking)	≈49.8%	≈0.3% (100 cycles) ≤1% (±15% read voltage fluctuation)	Fabricated devices with external instruments	[96]
	~	≈50%	≤1.2% (0–125 °C in mode 1) ≤0.3% (0–125 °C in mode 2)	Software processing of measured data	[97]
	~	50.06 ± 6.45%	≈0% (100 cycles: <0.46%)	Integrated chip	[85]
Colum comparison	Cu/pV3D3/Al	53.9 ± 8.0%	~		[99]
	TiO_x_/Al_2_O_3_	≈50% (128 × 128 array)	≤0.2% (128 × 128 array)	Software processing of measured data	[100]
	~	≈50%	≈0.1%	Simulation	[101]
	~	≈50.2%	≈10% (32 × 2 array)	Simulation	[103]
Delay comparison	~	≈49.9%	≈1% (0–80 °C) ≈0.75% (0.85–1.15 V)	Simulation	[105]
	~	≈49.4%	≈0.8% (0–80 °C) ≈3.8% (0.85–1.15 V)	Simulation	[107]
	~	≈51.8%	≈3.4% (10–30 °C: 0%)	Simulation	[111]
Threshold comparison	W/Ag/MgO/Ag/W	≈49.1%	0% (baking at 80 °C for 10 h)	Fabricated devices with external instruments	[113]
	TiN/TaO_x_/HfO_x_/TiN	≈50.5%	0% (100 °C: 4.5%)	Integrated chip	[114]
	~	≈47.9%	≤9% (0–85 °C) ≤12.4% (1.4–1.6 V)	Simulation	[118]

## Data Availability

No new data were created or analyzed in this study. Data sharing is not applicable to this article.
